# Asymmetric synthesis of fluorinated derivatives of aromatic and γ-branched amino acids via a chiral Ni(II) complex

**DOI:** 10.3762/bjoc.21.52

**Published:** 2025-03-21

**Authors:** Maurizio Iannuzzi, Thomas Hohmann, Michael Dyrks, Kilian Haoues, Katarzyna Salamon-Krokosz, Beate Koksch

**Affiliations:** 1 Institute of Chemistry and Biochemistry, Freie Universität Berlin, Arnimallee 20, 14195 Berlin, Germany

**Keywords:** chiral nickel complexes, fluorinated amino acids, gram-scale amino acid synthesis, stereoselective synthesis

## Abstract

Fluorinated amino acids are essential building blocks in the spheres of protein engineering and medicinal chemistry. In the last decades, a large number of different synthetic strategies have been developed to produce a large variety of fluorinated amino acids. Still, obtaining fluorinated amino acids in great quantities can be challenging, or the corresponding pathways are heavily time-consuming and synthetically challenging. In this context, chiral Ni(II) complexes can be powerful tools to obtain tailor‑made non‑canonical amino acids. In this work, we wanted to take advantage of this strategy and extend the range of this method to include additional fluorinated amino acids. We synthesized two fluorinated analogs of phenylalanine, which are still unexplored in the context of peptide and protein chemistry. Furthermore, both diastereomers of trifluoroleucine were synthesized, demonstrating that the described strategy can also be applied to synthesize enantio‑ and diastereomerically pure γ‑branched fluorinated amino acids. This work further underlines the importance of chiral Ni(II) complexes in the synthesis of fluorinated amino acids.

## Introduction

Non-natural amino acids are pivotal in protein engineering and drug development. Over 30% of approved small‑molecule drugs today contain non‑canonical amino acid building blocks [1–2]. In peptide and protein engineering, non‑natural amino acids significantly increase the respective range of tools used to modify a series of peptide and protein-related properties such as stability, specificity, and folding. In this regard, fluorinated amino acids are particularly important. Incorporation of fluorinated groups into the sequence of peptides and proteins can, for instance, regulate the respective hydrophobicity, alter the folding properties, and improve cell permeability [3–5].

**Scheme 1:**
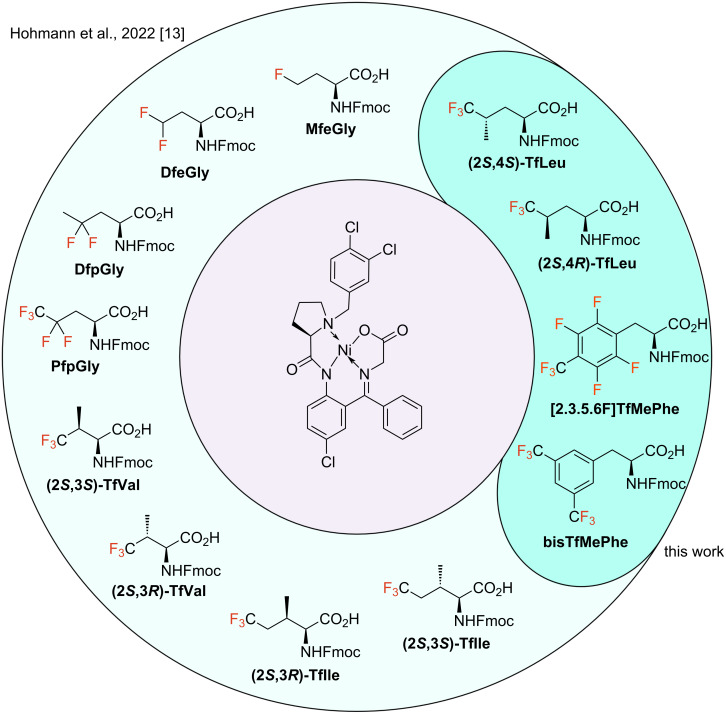
Previous work for obtaining different fluorinated amino acids and target fluorinated amino acids described in the context of this work.

Despite the evidence that unnatural amino acids play a significant role in the mentioned areas, synthesizing these building blocks can still be a major challenge [6]. A potent strategy in this regard is the utilization of chiral nickel complexes. In recent years, the Soloshonok working group demonstrated the synthesis of non‑natural amino acids using the corresponding chiral Ni(II) complex [7]. In addition to the high enantiomeric purity of the corresponding products, the scale of the reaction, which extends into the hectogram range, is a major strength of this method. In this context, Han et al. could show that the trifluorinated variant of α-aminobutyric acid, trifluoroethylglycine (TfeGly), can be synthesized on a 100 g scale with great enantiomeric purity [8]. The critical step here is the alkylation of the Ni(II) complex with the corresponding fluorinated alkyl iodide. The aryl moiety of the Ni(II) complex blocks the top face of the complex, ensuring the high diastereoselectivity of this transformation. In principle, synthesis on a gram-scale permits the study of highly fluorinated systems. Recently, we introduced fluoropeptides consisting nearly exclusively of fluorinated building blocks, which could only be accomplished by having the corresponding fluorinated amino acids in gram quantities [9–10]. In the last decades, others have also demonstrated that chiral Ni(II) complexes can be used to synthesize fluorinated amino acids [11–12]. Recently, our working group presented the synthesis of a broad range of fluorinated derivatives of different canonical and non-canonical amino acids (Scheme 1) [13]. Besides the linear fluorinated versions of α‑aminobutyric acid and norvaline, the β‑branched fluorinated amino acids such as trifluorovaline and trifluoroisoleucine were synthesized on a gram‑scale with excellent enantiomeric purities.

In this work, we increase the scope of this methodology even further. First, we present the synthesis of two fluorinated, aromatic amino acids: (2,3,5,6)-tetrafluoro-4-trifluoromethylphenylalanine ([2.3.5.6F]TfMePhe, **2**) and bis(trifluoromethyl)phenylalanine (bisTfMePhe, **3**) (Scheme 1). Neither amino acid has, to our knowledge, been described in the context of peptide chemistry yet. In general, fluorinated aromatic amino acids are essential building blocks that allow a modification of aromatic–aromatic interactions. For example, our group highlighted the influence of different fluorinated phenylalanine analogs on the aggregation rate of amyloid-forming NFGAIL peptides [14]. Amino acids **2** and **3**, with their respective fluorination pattern, might be fascinating compounds in this context. As already stated, chiral Ni(II) complexes can be used to synthesize fluorinated analogs of aliphatic canonical amino acids. Recently Naulet et al. presented a strategy for the synthesis of hexafluoroleucine (HfLeu) using a slightly modified version of the respective Ni(II) complex [12]. Furthermore, we introduce our attempts to synthesize the trifluorinated derivatives of leucine: (2*S*,4*S*)-trifluoroleucine ((2*S*,4*S*)-TfLeu) and (2*S*,4*R*)-trifluoroleucine ((2*S*,4*R*)-TfLeu) (Scheme 1). Here, we were especially interested in exploring the applicability of the Ni-based strategy to the synthesis of γ-branched amino acids.

## Results and Discussion

### Aromatic amino acids

**Scheme 2:**
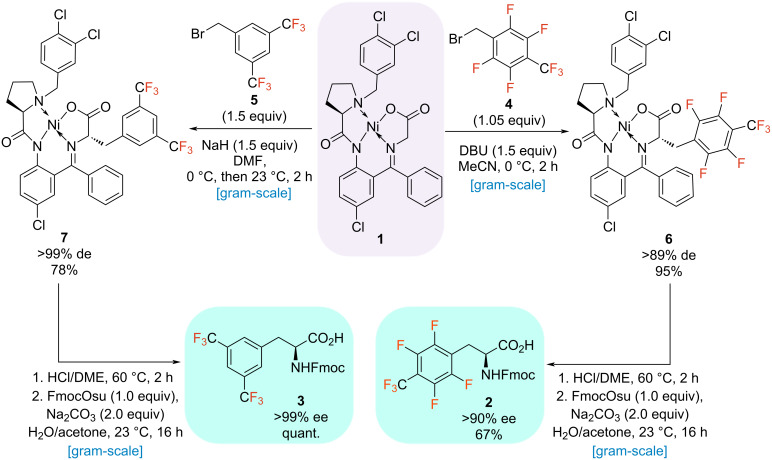
Synthesis of fluorinated aromatic amino acids **2** and **3**.

First, we concentrated our efforts on synthesizing the aromatic, fluorinated amino acids (Scheme 2). The corresponding alkyl bromide precursors were commercially available. We screened a broad range of reaction conditions such as temperature, base, solvent, and reagent equivalents to optimize the alkylation reaction for both bromides **4** and **5** (Table 1 and Table 2). For Ni(II) complex of [2.3.5.6F]TfMePhe (**6**), firstly we screened different inorganic and organic bases (Table 1, entries 1–3) and 1,8-diazabicyclo[5.4.0]undec-7-ene (DBU) was identified as optimal delivering a yield of alkylated complex of 60% (Table 1, entry 4). With DBU as base different solvents differing in polarity have been tested. At room temperature, acetonitrile proved best and increased the yield to 88% (Table 1, entry 11). Lowering the temperature to 0 °C led to a final yield of 94%. At these conditions (DBU, MeCN and 0 °C) the base and bromide equivalents were further modified but no further increase in yield could be achieved. Thus, 1.5 equiv DBU with 1.05 equiv alkyl bromide in MeCN at 0 °C have been identified as optimal conditions for the Ni complex formation (Table 1, entry 12). By employing these conditions, the reaction was carried out on a decagram-scale, and the respective alkylated Ni(II) complex **6** was isolated with an excellent yield of 95% and high diastereomeric purity of 90% de (Scheme 2).

**Table 1:** Optimization of the reaction conditions for the alkylation step using bromide **4**.

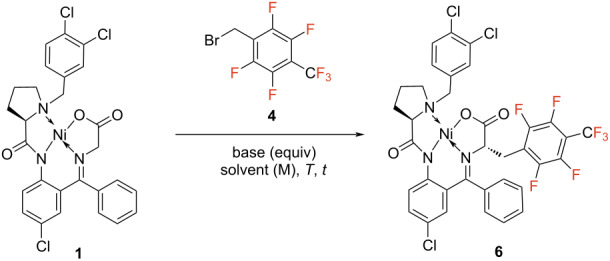							

Entry	Base	Base [equiv]	Alkyl bromide [equiv]	*T* [°C]	Solvent	Solvent [M]	Yield [%]^a^

1	NaH	1.50	1.05	0–rt	DMF	0.50	56
2	KOH/MeOH	1.50	1.05	0–rt	DMF	0.50	51
3	KO*t-*Bu	1.50	1.05	0–rt	DMF	0.50	41
4	DBU	1.50	1.05	0–rt	DMF	0.50	60
5	DBU	1.50	1.05	0–rt	NMP	0.50	60
6	DBU	1.50	1.05	0–rt	THF	0.50	57
7	DBU	1.50	1.05	0–rt	DMI	0.50	64
8	DBU	1.50	1.05	0–rt	MeCN	0.50	70
9	DBU	1.50	1.05	0–rt	MeCN	0.30	63
10	DBU	1.50	1.05	0–rt	MeCN	0.70	69
11	DBU	1.50	1.05	rt	MeCN	0.50	88
**12**	**DBU**	**1.50**	**1.05**	**0**	**MeCN**	**0.50**	**94**
13	DBU	1.10	1.05	0	MeCN	0.50	90
14	DBU	2.00	1.05	0	MeCN	0.50	77
15	DBU	1.50	1.50	0	MeCN	0.50	72
16	DBU	1.50	2.00	0	MeCN	0.50	47

^a^Determined by ^19^F NMR using 2-chloro-4-fluorotoluene as an internal standard.

In contrast, the optimal conditions to obtain the Ni(II) complex of bisTfMePhe have differed significantly. Here, sodium hydride (NaH) was identified as optimal base leading to a yield of 85% when using DMF as solvent at 0 °C to room temperature (Table 2, entry 4). Testing different base equivalents, solvents, solvent mixtures and temperatures didn’t lead to any yield improvement (Table 2, entries 7–13). Herein, using dimethylformamide (DMF), different equiv of alkyl bromide were further screened resulting in a yield of 93%. Thus, 1.5 equiv NaH with 1.5 equiv alkyl bromide in DMF at 0 °C to room temperature have been identified as optimal conditions (Table 2, entry 16). With optimized conditions in hand, the respective alkylated Ni(II) complex was isolated in a good yield of 78% and with excellent diastereomeric purity of >99% de (Scheme 2). Both alkylated Ni(II) complexes (**6** and **7**) were hydrolyzed under standard conditions (HCl/DME 60 °C, 2 h), and the subsequent fluorenylmethoxycarbonyl (Fmoc) protection with FmocOsu led to the formation of the desired fluorinated amino acids. Here, Fmoc-[2.3.5.6F]TfMePhe (**2**) was isolated in a yield of 67% and a good enantiomeric purity of 90% ee. Fmoc-bisTfMePhe (**3**) was obtained even in a quantitative yield and with a great enantiomeric excess of >99% ee (Scheme 2).

**Table 2:** Optimization of the reaction conditions for the alkylation step using bromide **5**.

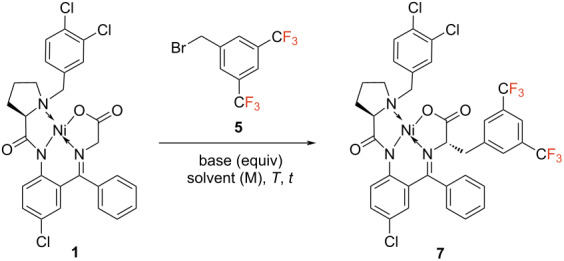							

Entry	Base	Base [equiv]	Alkyl bromide [equiv]	*T* [°C]	Solvent	Solvent [M]	Yield [%]^a^

1	DBU	1.50	1.05	0–rt	DMF	0.50	79
2	KOH/MeOH	1.50	1.05	0-rt	DMF	0.50	51
3	KO*t-*Bu	1.50	1.05	0–rt	DMF	0.50	59
4	NaH	1.50	1.05	0–rt	DMF	0.50	85
5	NaH	1.10	1.05	0–rt	DMF	0.50	70
6	NaH	2.00	1.05	0–rt	DMF	0.50	50
7	NaH	1.50	1.05	0	DMF	0.50	70
8	NaH	1.50	1.05	rt	DMF	0.50	55
9	NaH	1.50	1.05	0–rt	NMP	0.50	53
10	NaH	1.50	1.05	0–rt	THF	0.50	65
11	NaH	1.50	1.05	0–rt	THF/DMF	0.50	57
12	NaH	1.50	1.05	0–rt	MeCN	0.50	56
13	NaH	1.50	1.05	0–rt	DMI	0.50	61
14	NaH	1.50	1.05	0–rt	DMF	0.30	30
15	NaH	1.50	1.05	0–rt	DMF	0.70	48
**16**	**NaH**	**1.50**	**1.50**	**0–rt**	**DMF**	**0.50**	**93**
17	NaH	1.50	2.00	0–rt	DMF	0.50	27

^a^Determined by ^19^F NMR using 2-chloro-4-fluorotoluene as an internal standard.

### Trifluorinated derivatives of leucine

**Scheme 3:**
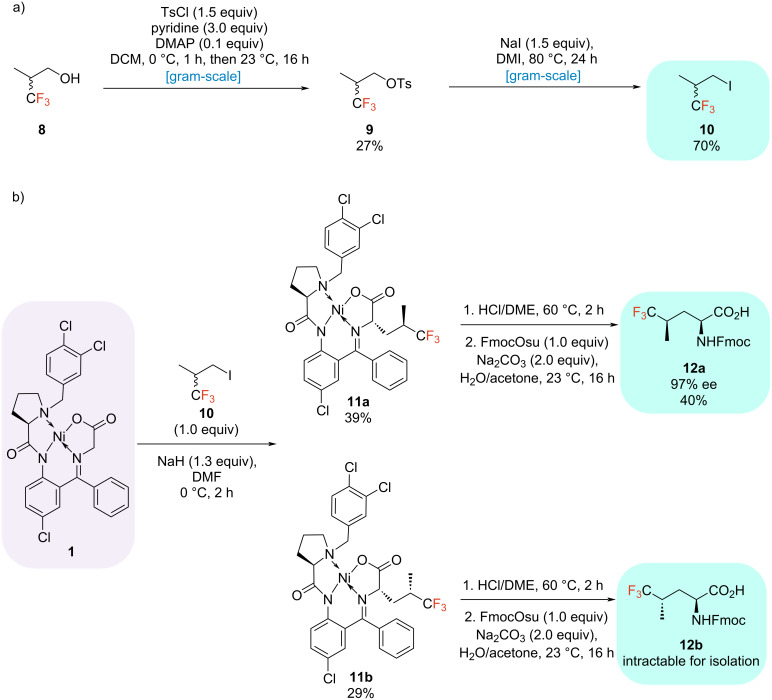
a) Gram-scale synthesis of fluorinated alkyl iodide precursor **10**; b) Synthesis of trifluorinated leucine analogs **12a** and **12b**.

The fluorinated alkyl iodide is commercially available but costly. Thus, we aimed at establishing an appropriate synthesis for this iodide. Our previous work showed that fluorinated alkyl iodides can be efficiently synthesized in gram-scale from the respective fluorinated alcohols using alkyl nonaflates as a key intermediate [13]. Based on these results, 3,3,3-trifluoro-2-methylpropan-1-ol (**8**) was selected as the starting material. We started our efforts by screening the reaction conditions to obtain the corresponding nonaflate. To our surprise, the corresponding yields of this transformation were rather unsatisfying (data not shown). Unfortunately, the yield could not be significantly improved by varying all essential reaction parameters. Therefore, a different strategy for the synthesis of alkyl iodides was investigated. A tosylate functionality was employed as a leaving group in the iodination reaction. Finally, the desired fluorinated tosylate **9** could be isolated on a gram-scale in a moderate yield of 27% (Scheme 3a). Subsequently, the iodination of the tosylate has been optimized regarding temperature and time and optimized conditions at 80 °C for 24 h (Table 3, entry 3) resulted in the synthesis of the desired fluorinated alkyl iodide **10** with a great yield of 87%. However, scaling up on gram-scale, a slightly decreased yield of 70% was achieved (Scheme 3a).

**Table 3:** Optimization of the reaction conditions for the synthesis of the alkyl iodide **10**.

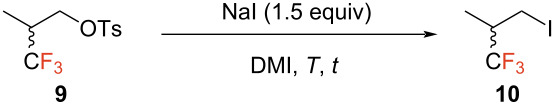			

Entry	*T* [°C]	Time [h]	Yield [%]^a^

1	100	24	0
2	60	24	40
**3**	**80**	**24**	**87**
4	80	72	87
5	80	3	31

^a^Determined by ^19^F NMR using 2-chloro-4-fluorotoluene as an internal standard.

With the fluorinated alkyl iodide precursor **10**, the corresponding alkylation reaction with the Ni(II) complex **1** was conducted under previously optimized conditions for the synthesis of Fmoc-TfIle [13] in terms of base (NaH) and solvent (DMF) and thoroughly screened in terms of base equivalents, concentration and temperature (Table 4).

**Table 4:** Optimization of the reaction conditions for the synthesis of alkylated complexes **11a** and **11b**.

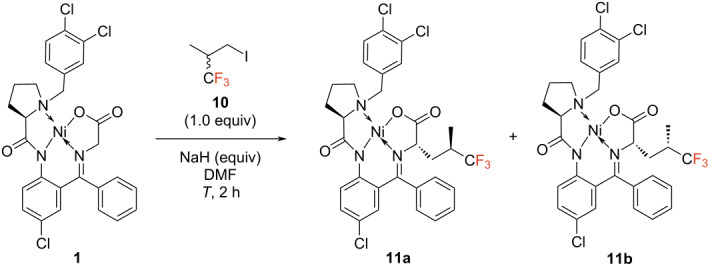				

Entry	NaH [equiv]	DMF [mL/mmol of complex]	*T* [^o^C]	Yield [%]^a^

**1**	**1.3**	**5**	**0**	**62**
2	1.3	5	rt	31
3	1.3	5	−10	20
4	1.3	2.50	rt	7
5	1.3	2.50	0	41
6	3	5	rt	24
7	3	5	0	33
8	3	2.50	rt	36
9	3	2.50	0	31

^a^Determined by ^19^F NMR using 2-chloro-4-fluorotoluene as an internal standard.

By adjusting the base to 1.3 equivalents and carrying the reaction at 0 °C, the yield of the transformation could be improved from 30% to 62% (Table 4, entry 1). By applying the respective conditions, both Ni(II) complexes of TfLeu were synthesized with an excellent overall total alkylation yield of 68%. Since two desired diastereomers are formed in this case, they had to be subsequently separated from one another. This was accomplished using flash column chromatography. The respective Ni(II) complexes of TfLeu (**11a**, **11b**) could be isolated with excellent diastereomeric purities of 95 and 97% de. Both alkylated Ni(II) complexes were then hydrolyzed under standard conditions (HCl/DME 60 °C, 2 h), and the subsequent fluorenylmethoxycarbonyl (Fmoc) protection with FmocOsu led to the formation of the desired fluorinated amino acids. However, only one isomer could be isolated, requiring several chromatographic purification steps (EtOAc/*n*-pentane, 2% AcOH). Hence, in the here described case, the final hydrolysis and Fmoc protection resulted the enantiomerically pure isomer Fmoc-(2*S,*4*R*)-TfLeu (**12a**), isolated in a good yield of 40% and with excellent enantiomeric purity of 97% ee. The configuration was determined by analyzing the ^1^H NMR spectrum in the δ = 2.8–1.7 ppm region, which displayed the characteristic diastereotopic hydrogen atoms of this compound, as previously reported by Biava et al. [15]*.* Unfortunately, the corresponding (2*S*,4*S*)-isomer **12b** was intractable for isolation (yield < 5%) despite numerous attempts.

## Conclusion

This work described the stereoselective and gram-scale synthesis of a highly attractive palette of fluorinated amino acids prepared for application in solid-phase peptide synthesis. First, two different fluorinated variants of phenylalanine were synthesized, which have yet to be described in the context of peptide chemistry. The several CF_3_ groups employ a strong inductive effect on the aromatic ring structure, making these amino acids highly interesting building blocks for modification of aromatic–aromatic interactions in the context of protein folding, interaction and function. Furthermore, the synthesis of both isomers of trifluoroleucine was described. (2*S*,4*R*)*-*Trifluoroleucine could be isolated on a milligram-scale in good yields and excellent enantiomeric purities, representing a viable synthetic route for these building blocks adding another strategy to the repertoire of published syntheses for this fluorinated derivative of natural leucine. Further attempts to the isolation of the second isomer will be made. Overall, this work further underlines the potential of chiral nickel complexes in synthesizing fluorinated amino acids. The diverse range of fluorinated amino acids that can be synthesized from a single starting material is a unique feature of this method, making it an important cornerstone of fluoropeptide chemistry.

## Experimental

### General information

Air- and hydrolysis-sensitive reactions were carried out under exclusion of air and water in Schlenk vessels at a Schlenk unit/oil pump vacuum under nitrogen atmosphere. The stated reaction temperatures are the respective values of the silicone oil heating bath. All reactions were stirred with an electric magnetic stirrer. ^1^H, ^13^C, and ^19^F NMR spectra were measured at room temperature with a JEOL ECP 600 (JEOL, Tokyo, Japan) device. MestReNova Version 10.0.0 (Mestrelab Research S. L., Santiago de Compostela, Spain) was used to analyze the respective spectra. The chemical shifts are given in parts per million (ppm). The ^1^H and ^13^C NMR chemical shifts were referenced against the specific internal solvent residual peaks (CDCl_3_, CD_3_OD) and given to tetramethylsilane as internal standard (δ = 0.00 ppm). High-resolution mass spectra (HRMS) of the obtained compounds were measured on an Agilent 6220 ESI-TOF MS instrument (Agilent Technologies, Santa Clara, CA, USA) using a spray voltage of 4 kV. The prepared samples were injected into the spray chamber using a syringe pump with flow rates of 10 to 40 μL/min. The desolvation gas was adjusted to 15 psi. Other parameters were optimized for maximal abundance of [M + H]^+^, [M + Na]^+^, or [M + K]^+^. High-resolution electron ionization mass spectra (HREIMS) were measured on a MAT 711 (Varian MAT, Bremen, Germany). Electron energy for EI was set to 70 eV. Infrared spectra (IR) were measured on an ALPHA II (Bruker, Billerica, USA) spectrometer. Characteristic absorption bands are given in wave numbers (cm^−1^). Qualitative thin-layer chromatography (TLC) was carried out on aluminum plates coated with silica gel 60 F254 (Merck, Darmstadt, Germany). The TLC plates were analyzed using UV light at 254 nm. Flash chromatography was performed on silica gel 60 M from Macherey-Nagel (grain size of 40−63 μm). The conditions are given in the form “(A/B = a:b)”, where A/B refers to the solvents used as mobile phase and a:b to their volume ratio. Analytical high-performance liquid chromatography (HPLC) was used to determine the purity of the obtained Fmoc-protected amino acids. The respective HPLC runs were carried out on a Primaide DAD system (VWR/Hitachi, Germany). The system works with a low-pressure gradient containing a HPLC pump (1110) with a 6-channel solvent degaser, an organizer, an autosampler (1210) with a 100 μL sample loop, a column oven (1310) and a diode array detector (1430). A Kinetex C18 (2) column (5 μm, 250 Å × 4.6 mm, Phenomenex, Torrance, CA, USA) was used. H_2_O and MeCN, both containing 0.1% (v/v) TFA, served as eluents. Fmoc-protected amino acids were detected at 220 nm. For a chiral analysis, a CHIRALPAK ZWIX(−) column ((R,R)-ACHSA immobilized on 3 μm silica gel, 250 × 4 mm, Chiral Technologies Europe, Illkirch Cedex, France) was used. MeCN/MeOH/H_2_O mixture with 50 mM formic acid and 25 mM diethylamine was used as eluent. A flow rate of 0.5 mL/min was applied and the detection of the respective compounds occurred at 280 nm. Data analysis was carried out with the EZChrom ELITE software (version 3.3.2 SP2, Agilent). Ni(II) complex **1** was synthesized according the procedure described by Romoff et al. [16]. Sodium hydride was used as a 60% dispersion in mineral oil. Triethylamine was dried over CaH_2_ and distilled freshly before use. Perfluorobutanesulfonyl fluoride was dried over CaCl_2_ and freshly distilled before use. Other chemicals were used without further purification and obtained from commercial sources.

### Synthesized compounds

**3,3,3-Trifluoro-2-methylpropyl 4-methylbenzene-1-sulfonate (9): 8** (5.00 g, 39.0 mmol, 1.0 equiv), was dissolved in DCM (48.8 mL). *p*-Toluenesulfonyl chloride (11.15 g, 58.5 mmol, 1.5 equiv) and DMAP (0.48 g, 3.9 mmol, 0.1 equiv) were added and the mixture was cooled to 0 °C before pyridine (9.41 mL, 117.0 mmol, 3.0 equiv) was added. After 1 h of stirring, the reaction was warmed to room temperature and further stirred for 16 h. Subsequently, the mixture was diluted with H_2_O (25 mL) and extracted with DCM (2 × 40 mL). The combined organic phases were then washed with aq HCl (2 M, 12 mL), saturated aqueous NaHCO_3_ solution (12 mL), and brine (12 mL). Afterward, the combined organic phases were dried over Na_2_SO_4_, filtered, and dried in vacuo for 16 h. The crude product was purified via flash-column chromatography (*n*-Pen/EtOAc, 10:1). The product **9** was obtained as a colorless oil (2.99 g, 10.6 mmol, 27%). ^1^H NMR (600 MHz, CDCl_3_) δ 7.81–7.76 (m, 2H), 7.39–7.34 (m, 2H), 4.16 (dd, *J* = 10.3, 5.3 Hz, 1H), 3.97 (dd, *J* = 10.3, 6.8 Hz, 1H), 2.62–2.54 (m, 1H), 2.46 (s, 3H), 1.23–1.15 (m, 3H) ppm; ^19^F NMR (565 MHz, CDCl_3_) δ −71.53 (d, J = 9.4 Hz, 3F) ppm.

**1,1,1-Trifluoro-3-iodo-2-methylpropane (10):** A mixture of **9** (7.44 g, 26.4 mmol, 1.0 equiv) and 1,3-dimethyl-2-imidazolinone (DMI) (8.8 mL) was heated to 80 °C before NaI (5.94 g, 39.6 mmol, 1.5 equiv) was added and the reaction mixture was stirred for 24 h. The crude product was purified by vacuum distillation (4 × 10^−2^ mbar). The product **10** was obtained as colorless liquid (4.41 g, 18.5 mmol, 70%). ^1^H NMR (600 MHz, CDCl_3_) δ 3.44 (dd, *J* = 10.3, 3.5 Hz, 1H), 2.97 (t, *J* = 10.1 Hz, 1H), 2.53–2.43 (m, 1H), 1.29 (d, *J* = 6.9 Hz, 3H) ppm; ^19^F NMR (565 MHz, CDCl_3_) δ −72.74 (d, *J* = 7.9 Hz) ppm.

**Ni(II)-Schiff base complex of [2.3.5.6F]TfMePhe 6:** Under inert conditions and at 0 °C, DBU (2.23 mL, 15.0 mmol, 1.5 equiv) was added dropwise to a mixture of **1** (6.0 g, 9.97 mmol, 1.0 equiv) and **4** (1.77 mL, 10.5 mmol, 1.05 equiv) in dry, vented MeCN (20 mL). After 2 h of stirring at 0 °C, H_2_O (30 mL) was added. After 1 h of stirring, the precipitated product was filtered, washed with H_2_O (15 mL), and dried in vacuo at 60 °C to give **6** (7.81 g, 9.39 mmol, 95%, 91% purity determined by analytical HPLC) as an orange solid. The crude was used without further purification. ^1^H NMR (600 MHz, CDCl_3_) δ 8.88 (d, *J* = 2.2 Hz, 1H), 8.07 (d, *J* = 9.3 Hz, 1H), 7.79 (dd, *J* = 8.2, 2.2 Hz, 1H), 7.62 (p, *J* = 5.7 Hz, 2H), 7.49 (dt, *J* = 9.0, 4.4 Hz, 1H), 7.37 (d, *J* = 8.1 Hz, 1H), 7.33 (dt, *J* = 6.3, 2.0 Hz, 1H), 7.15 (dd, *J* = 9.3, 2.8 Hz, 1H), 6.88 (d, *J* = 7.7 Hz, 1H), 6.63 (d, *J* = 2.8 Hz, 1H), 4.30 (d, *J* = 12.7 Hz, 1H), 4.13 (dd, *J* = 10.1, 5.9 Hz, 1H), 3.83 (dd, *J* = 13.4, 10.0 Hz, 1H), 3.58 (dt, *J* = 12.7, 3.4 Hz, 1H), 3.55–3.49 (m, 1H), 3.39 (dt, *J* = 11.0, 6.1 Hz, 1H), 3.22 (d, *J* = 12.7 Hz, 1H), 3.08 (dd, *J* = 13.4, 6.0 Hz, 1H), 2.78–2.58 (m, 1H), 2.37–2.24 (m, 1H), 2.08 (td, *J* = 11.0, 5.9 Hz, 1H), 1.65 (m, 1H); ^13^C NMR (151 MHz, CDCl_3_) δ 180.1, 176.4, 171.6, 144.7, 141.1, 134.9 (2C), 133.8, 133.7 (2C), 133.6, 133.1, 132.5, 132.2 (2C), 131.3, 130.9, 130.1, 129.8, 129.6, 127.5 (2C), 127.3 (2C), 127.3, 126.1, 124.4 (2C), 71.5, 68.3, 63.2, 58.7, 31.0, 29.1, 23.9; ^19^F NMR (565 MHz, CDCl_3_) δ −56.23 (t, *J* = 20.7 Hz), −140.45 (dq, *J* = 28.6, 11.9, 11.1 Hz); ATR-FTIR (neat): 3587, 2975, 2868, 1634, 1500, 1333, 1249, 1137, 870, 820, 709 cm^−1^; HRMS (ESI-TOF) *m*/*z*: [M + Na]^+^ calcd for C_35_H_23_Cl_3_F_7_N_3_NiO_3_, 853.9916; found, 853.9958.

**Ni(II)-Schiff base complex of bisTfMePhe 7:** Under inert conditions and at 0 °C, NaH (1.0 g, 24.9 mmol, 1.5 equiv) was added slowly to a mixture of **1** (10.0 g, 16.6 mmol, 1.0 equiv) and **5** (4.64 mL, 24.9 mmol, 1.5 equiv) in dry, vented DMF (19.9 mL). The reaction was stirred at room temperature for 2 h. Afterwards, H_2_O (50 mL) was added. After 1 h of stirring, the precipitated product was filtered, washed with H_2_O (20 mL), and dried in vacuo at 60 °C. The crude material was purified by flash column chromatography (SiO_2_, 10 **→** 100% EtOAc in diethyl ether) to yield **7** as a red solid (10.7 g, 12.9 mmol, 78%, 99% purity determined by analytical HPLC). ^1^H NMR (600 MHz, CDCl_3_) δ 8.90 (d, *J* = 2.2 Hz, 1H), 8.18 (d, *J* = 9.3 Hz, 1H), 7.81 (s, 1H), 7.66 (dd, *J* = 8.2, 2.2 Hz, 1H), 7.67–7.59 (m, 2H), 7.55–7.47 (m, 1H), 7.37 (dt, *J* = 6.6, 1.8 Hz, 1H), 7.34 (d, *J* = 8.2 Hz, 1H), 7.26 (d, *J* = 1.5 Hz, 2H), 7.15 (dd, *J* = 9.3, 2.6 Hz, 1H), 6.89 (dt, *J* = 7.7, 1.0 Hz, 1H), 6.64 (d, *J* = 2.6 Hz, 1H), 4.25 (d, *J* = 12.7 Hz, 1H), 4.20 (dd, *J* = 7.8, 4.0 Hz, 1H), 3.41–3.32 (m, 1H), 3.30 (dd, *J* = 11.1, 5.9 Hz, 1H), 3.18 (dd, *J* = 13.8, 7.8 Hz, 1H), 3.17 (d, *J* = 12.7 Hz, 1H), 3.10 (dd, *J* = 13.7, 3.9 Hz, 1H), 2.86 (dddd, *J* = 19.2, 14.0, 6.6, 3.8 Hz, 1H), 2.49 (dddd, *J* = 13.7, 11.0, 9.6, 8.3 Hz, 1H), 2.45–2.34 (m, 1H), 2.07–2.01 (m, 1H), 1.99 (dt, *J* = 11.1, 5.4 Hz, 1H); ^13^C NMR (151 MHz, CDCl_3_) δ 180.1, 177.4, 171.4, 141.3, 138.3, 134.9, 133.8, 133.7, 133.5, 133.1, 133.1, 132.4, 132.1, 131.9, 131.2, 130.9, 130.1, 130.0, 129.9, 129.6, 127.5, 127.3, 126.9, 126.0, 124.3 (2C), 124.1, 122.3, 121.5, 71.2, 70.9, 63.3, 58.6, 40.7, 30.8, 23.7; ^19^F NMR (565 MHz, CDCl_3_) δ −62.57 (s); ATR-FTIR (neat): 3062, 2982, 2868, 1638, 1463, 1278, 1133, 894, 820, 705 cm^−1^; HRMS (ESI-TOF) *m*/*z*: [M + Na]^+^ calcd for C_36_H_26_Cl_3_F_6_N_3_NiO_3_, 850.0167; found, 850.0181.

**Ni(II)-Schiff base complexes of (2*S*,4*R*)-TfLeu 11a, (2*S*,4*S*)-TfLeu 11b:** Under inert conditions and at 0 °C, NaH (0.13 g, 5.4 mmol, 1.3 equiv) was added slowly to a mixture of **1** (2.0 g, 4.16 mmol, 1.0 equiv), and **10** (1.0 g, 4.16 mmol, 1.0 equiv) in dry, vented DMF (5 mL). The reaction was stirred at 0 °C for 2 h. Afterwards, H_2_O (10 mL) was added. After 1 h of stirring, an additional amount of H_2_O (5 mL) was added, and the reaction mixture was stirred for further 1 h. Afterwards, the precipitated solid was filtered and washed with H_2_O/DMF (1:2, 20 mL). The resulting diastereomeric crude products were purified and separated via flash-column chromatography (chloroform/acetone, 3:1) to yield to **11a** (2*S*,4*R*) (1.18 g, 1.7 mmol, 39%) and **11b** (2*S*,4*S*) (0.87 g, 1.22 mmol, 29%) as red solids. **11a**: ^1^H NMR (600 MHz, CDCl_3_) δ 8.86 (s, 1H), 8.00 (d, *J* = 9.2 Hz, 1H), 7.75 (d, *J* = 8.2 Hz, 1H), 7.62–7.46 (m, 3H), 7.35 (d, *J* = 8.1 Hz, 1H), 7.30 (d, *J* = 7.4 Hz, 1H), 7.13–7.08 (m, 1H), 6.90 (d, *J* = 7.6 Hz, 1H), 6.59–6.56 (m, 1H), 4.32–4.25 (m, 1H), 3.80 (dd, *J* = 12.0, 3.9 Hz, 1H), 3.64 (dt, *J* = 19.7, 10.5 Hz, 1H), 3.52 (dt, *J* = 10.6, 5.9 Hz, 1H), 3.35 (dd, *J* = 11.4, 5.7 Hz, 1H), 3.19 (d, *J* = 12.6 Hz, 1H), 2.84 (t, *J* = 12.6 Hz, 1H), 2.72–2.50 (m, 3H), 2.27 (dt, *J* = 13.9, 7.1 Hz, 1H), 2.14 (s, 1H), 2.06 (dt, *J* = 11.6, 5.8 Hz, 1H), 1.41 (td, *J* = 12.6, 3.9 Hz, 1H), 0.44 (d, *J* = 6.8 Hz, 2H) ppm; ^13^C NMR (151 MHz, CDCl_3_) δ 180.07, 177.89, 170.35, 140.69, 134.95, 133.69, 133.51, 132.66, 132.46, 132.11, 131.17, 130.58, 130.01, 129.52, 128.74, 127.49, 127.29 (d, *J* = 4.4 Hz), 125.97, 124.48, 71.36, 67.22, 63.04, 58.61, 35.99, 34.58, 34.40, 30.96 (d, *J* = 15.8 Hz), 24.03, 23.17, 11.07 ppm; ^19^F NMR (565 MHz, CDCl_3_) δ −73.96 (d, *J* = 9.2 Hz) ppm.

**11b**: ^1^H NMR (600 MHz, CDCl_3_) δ 8.84 (s, 1H), 8.01 (d, *J* = 9.4 Hz, 1H), 7.76 (d, *J* = 8.2 Hz, 1H), 7.54 (q, *J* = 9.2 Hz, 2H), 7.47 (t, *J* = 7.6 Hz, 1H), 7.34 (d, *J* = 8.1 Hz, 1H), 7.26 (d, *J* = 6.9 Hz, 1H), 7.10 (d, *J* = 9.3 Hz, 1H), 6.85 (d, *J* = 7.6 Hz, 1H), 6.57 (s, 1H), 4.30 (d, *J* = 12.6 Hz, 1H), 3.92 (dd, *J* = 10.8, 4.8 Hz, 1H), 3.61 (q, *J* = 10.2 Hz, 1H), 3.52 (t, *J* = 8.4 Hz, 1H), 3.36 (dd, *J* = 11.5, 5.6 Hz, 1H), 3.19 (d, *J* = 12.6 Hz, 1H), 2.75–2.48 (m, 3H), 2.36–2.24 (m, 2H), 2.15 (s, 1H), 2.07–2.02 (m, 2H), 1.07 (d, *J* = 7.0 Hz, 3H) ppm; ^13^C NMR (151 MHz, CDCl_3_) δ 179.95, 178.16, 170.72, 140.60, 134.92, 133.80–133.43 (m), 132.63, 132.34 (d, *J* = 9.3 Hz), 131.18, 130.53, 130.00, 129.59 (d, *J* = 8.7 Hz), 128.50, 127.43, 127.22 (d, *J* = 19.1 Hz), 125.98, 124.25, 71.38, 68.54, 63.06, 58.50, 37.65, 34.44 (d, *J* = 27.1 Hz), 34.08 (d, *J* = 26.7 Hz), 30.90, 24.12, 14.86 ppm; ^19^F NMR (565 MHz, CDCl_3_) δ −73.28 (d, *J* = 9.0 Hz) ppm.

**(*S*)-2-((((9*H*-Fluoren-9-yl)methoxy)carbonyl)amino)-3-(2,3,5,6-tetrafluoro-4-(trifluoromethyl)phenyl)propanoic acid [tetrafluoro(trifluoromethyl)phenylalanine, [2.3.5.6F]TfMePhe] (2):** At room temperature, aq HCl (3 M, 15.4 mL, 46.3 mmol, 5.0 equiv) was added to a stirring solution of **6** (7.70 g, 9.26 mmol, 1.0 equiv) in DME (30.9 mL). The resulting mixture was stirred at 60 °C for 2 h. After cooling to room temperature, the precipitated ligand was filtered and washed with H_2_O (15 mL). The filtrate was concentrated under reduced pressure to a volume of 30 mL. Again, the precipitated ligand was filtered and washed with H_2_O (15 mL). Both fractions of the ligand were united and dried in vacuo at 40 °C. To the filtrate, MeCN (18.5 mL) and EDTA-Na_2_ (3.45 g, 9.26 mmol, 1.0 equiv) were added and the reaction mixture was stirred at room temperature for 2 h. Subsequently, the solution was treated with aq NaOH (18 M) to pH 7 and Na_2_CO_3_ (1.96 g, 18.5 mmol, 2.0 equiv) was added. FmocOSu (3.12 g, 9.26 mmol, 1.0 equiv) was dissolved in acetone (37.0 mL) and added dropwise to the reaction mixture. After 17 h of stirring at room temperature, MeCN and acetone were removed under reduced pressure, H_2_O (40 mL) was added, and the mixture was treated with aq HCl (6 M) to pH 2. The resulting solution was extracted with EtOAc (4 × 40 mL), dried over Na_2_SO_4_, filtered, and concentrated in vacuo. The crude material was purified by flash column chromatography (SiO_2_, 0 **→** 10% MeOH in DCM) to yield **2** as a yellow solid (3.36 g, 9.26 mmol, 67%, 91% purity determined by analytical HPLC). ^1^H NMR (600 MHz, CDCl_3_) δ 7.77 (d, *J* = 7.6 Hz, 2H), 7.64–7.55 (m, 2H), 7.37 (tt, *J* = 7.6, 0.9 Hz, 2H), 7.28 (td, *J* = 7.4, 1.2 Hz, 2H), 4.65 (d, *J* = 4.7 Hz, 1H), 4.51 (dd, *J* = 9.3, 5.5 Hz, 1H), 4.34–4.20 (m, 2H), 4.14 (t, *J* = 7.0 Hz, 1H), 3.42 (dd, *J* = 14.0, 5.5 Hz, 1H), 3.33–3.23 (m, 1H); ^13^C NMR (151 MHz, CDCl_3_) δ 173.3, 158.4, 148.0 (2C), 146.2 (2C), 145.1 (2C), 142.6 (2C), 128.8 (2C), 128.1 (2C), 126.1 (2C), 123.3, 120.9 (3C), 108.9, 68.2, 54.1, 48.2, 26.9; ^19^F NMR (565 MHz, CDCl_3_) δ −57.00 (t), −141.58 (td), −142.21 to −144.04 (m); ATR-FTIR (neat): 3313, 3066, 2945, 1698, 1492, 1263, 1146, 966, 738 cm^−1^; HRMS (ESI-TOF) *m*/*z*: [M − H]^−^ calcd for C_25_H_15_F_7_NO_4_, 526.0894; found, 526.0871.

**(*S*)-2-((((9*H*-Fluoren-9-yl)methoxy)carbonyl)amino)-3-(3,5-bis(trifluoromethyl)phenyl)propanoic acid [bis(trifluoromethyl)phenylalanine, bisTfMePhe] (3):** At room temperature, aq HCl (3 M, 21.4 mL, 64.0 mmol, 5.0 equiv) was added to a stirred solution of **7** (10.6 g, 12.8 mmol, 1.0 equiv) in DME (42.7 mL). The resulting mixture was stirred at 60 °C for 2 h. After cooling to room temperature, the precipitated ligand was filtered and washed with H_2_O (20 mL). The filtrate was concentrated under reduced pressure to a volume of 40 mL. Again, the precipitated ligand was filtered and washed with H_2_O (20 mL). Both fractions of the ligand were united and dried in vacuo at 40 °C. To the filtrate, MeCN (25.6 mL) and EDTA-Na_2_ (4.77 g, 12.8 mmol, 1.0 equiv) were added and the reaction mixture was stirred at room temperature for 2 h. Subsequently, the solution was treated with aq NaOH (18 M) to pH 7 and Na_2_CO_3_ (2.71 g, 25.6 mmol, 2.0 equiv) was added. FmocOSu (4.32 g, 12.8 mmol, 1.0 equiv) was dissolved in acetone (51.2 mL) and added dropwise to the reaction mixture. After 17 h of stirring at room temperature, MeCN and acetone were removed under reduced pressure, H_2_O (50 mL) was added, and the mixture was treated with aq HCl (6 M) to pH 2. The resulting solution was extracted with EtOAc (4 × 50 mL), dried over Na_2_SO_4_, filtered, and concentrated in vacuo. The crude material was purified by flash column chromatography (SiO_2_, 20 **→** 100% EtOAc in diethyl ether) to yield **3** as an off-white solid (6.90 g, 12.8 mmol, 100%, 84% purity determined by analytical HPLC). ^1^H NMR (600 MHz, CDCl_3_) δ 7.91–7.85 (m, 2H), 7.82 (s, 1H), 7.76 (ddd, *J* = 7.7, 2.4, 1.5 Hz, 2H), 7.61–7.52 (m, 2H), 7.36 (td, *J* = 7.5, 4.0 Hz, 2H), 7.25 (tdd, *J* = 7.4, 2.8, 1.1 Hz, 2H), 4.48 (dd, *J* = 9.7, 4.9 Hz, 1H), 4.33–4.19 (m, 2H), 4.14 (t, *J* = 7.4 Hz, 1H), 3.41 (dd, *J* = 14.2, 4.9 Hz, 1H), 3.12 (dd, *J* = 14.0, 9.7 Hz, 1H); ^13^C NMR (151 MHz, CDCl_3_) δ 174.7, 158.4, 145.2 (2C), 142.5 (2C), 142.5, 132.6 (2C), 131.0 (2C), 128.7 (2C), 128.1 (2C), 126.2 (2C), 125.8, 124.0, 121.5, 120.9 (2C), 68.1, 56.4, 48.3, 38.12; ^19^F NMR (565 MHz, CDCl_3_) δ −63.42 (s): ATR-FTIR (neat): 3305, 3066, 2971, 1681, 1451, 1278, 1119, 983, 734 cm^−1^; HRMS (ESI-TOF) *m*/*z*: [M + Na]^+^ calcd for C_26_H_19_F_6_NO_4_, 546.1116; found, 546.1080.

**(2*S*,4*R*)-2-((((9*H*-Fluoren-9-yl)methoxy)carbonyl)amino)-5,5,5-trifluoro-4-methylpentanoic acid [Trifluoroleucine, TfLeu] (12a):** At room temperature aq HCl (3 M, 2.70 mL, 8 mmol, 5 equiv) was added to a stirring solution of **11a** (1.21 g, 1.60 mmol, 1.0 equiv) in DME (5.33 mL). The resulting mixture was stirred at 60 °C for 2 h. After cooling at room temperature, the precipitated ligand was filtered and washed with H_2_O (10 mL) leading to further precipitation of ligand. Again, the precipitated was filtered and washed with H_2_O (5 mL). Both fractions of the ligand were combined and dried in vacuo at 40 °C. To the filtrate, MeCN (3.20 mL) and EDTA-Na_2_ (0.54 g, 1.60 mmol, 1.0 equiv) were added and the reaction mixture was stirred at room temperature for 2 h. Subsequently, the solution was treated with aq NaOH (48%) to pH 8 and Na_2_CO_3_ (0.34 g, 3.20 mmol, 2.0 equiv) was added. FmocOSu (0.54 g, 1.60 mmol, 1.00 equiv) was dissolved in acetone (6.40 mL) and added dropwise to the reaction mixture. After 17 h of stirring at room temperature, MeCN and acetone were removed under reduced pressure. The mixture was treated with aq HCl (2 M) to pH 2. The resulting solution was extracted with EtOAc (6 × 40 mL), dried over Na_2_SO_4_, filtered, and concentrated in vacuo. The crude material undergone two purification steps by flash column chromatography (SiO_2_, EtOAc/*n*-Pen, 2% AcOH) to yield **12 a** as a white solid (0.26 g, 0.64 mmol, 40%) according to literature [15]. ^1^H NMR (600 MHz, CD_3_OD): δ 7.78–7.73 (m, 2H), 7.67–7.61 (m, 2H), 7.35 (tq, *J* = 7.5, 1.0 Hz, 2H), 7.27 (td, *J* = 7.5, 1.2 Hz, 2H), 4.85 (s, 2H), 4.38–4.33 (m, 2H), 4.26–4.16 (m, 2H), 2.44–2.36 (m, 1H), 2.19 (dt, *J* = 14.3, 6.2 Hz, 1H), 1.64 (ddd, *J* = 14.3, 8.6, 7.1 Hz, 1H), 1.13 (d, *J* = 7.0 Hz, 2H) ppm; ^13^C NMR (151 MHz, CD_3_OD) δ 173.65, 157.23, 144.00, 143.81, 141.29, 129.28, 127.45, 126.82 (d, *J* = 4.3 Hz), 124.90, 119.58 (d, *J* = 3.1 Hz), 66.58, 52.13, 47.10, 35.12 (q, *J* = 26.5 Hz), 31.73, 12.43 (d, *J* = 3.7 Hz) ppm; ^19^F NMR (565 MHz, CD_3_OD) δ −74.50 (d, *J* = 8.2 Hz) ppm; ATR-FTIR (neat): 3392, 3022, 2920, 1698, 1522, 1446, 1262, 1170, 1050, 740 cm^−1^; HRMS (ESI-TOF) *m*/*z*: [M + Na]^+^ calcd for C_24_H_20_F_3_NO_4_, 430.1236; found, 430.1288.

## Supporting Information

File 1 NMR spectra and HPLC chromatograms.

## Data Availability

Data generated and analyzed during this study is available from the corresponding author upon reasonable request.
