# Supplementary material for: Asymmetric synthesis of fluorinated derivatives of aromatic and γ-branched amino acids via a chiral Ni(II) complex
Source: Beilstein J Org Chem. 2025 Mar 21;21:659–69. doi: 10.3762/bjoc.21.52 (PMC11931639; doi:10.3762/bjoc.21.52)
Supplement: File 1 — NMR spectra and HPLC chromatograms. [file Beilstein_J_Org_Chem-21-659-s001.pdf]

BEILSTEIN JOURNAL OF ORGANIC CHEMISTRY

# Supporting Information

for

# Asymmetric synthesis of fluorinated derivatives of aromatic and γ-branched amino acids via a chiral Ni(II) complex

Maurizio Iannuzzi, Thomas Hohmann, Michael Dyrks, Kilian Haoues,
Katarzyna Salamon-Krokosz and Beate Koksch

*Beilstein J. Org. Chem.* **2025,** *21,* 659–669. doi:10.3762/bjoc.21.52

# NMR spectra and HPLC chromatograms

License and Terms: This is a supporting information file under the terms of the Creative Commons Attribution License (https://creativecommons.org/licenses/by/4.0). Please note that the reuse, redistribution and reproduction in particular requires that the author(s) and source are credited and that individual graphics may be subject to special legal provisions.
The license is subject to the *Beilstein Journal of Organic Chemistry* terms and conditions: (https://www.beilstein-journals.org/bjoc/terms)

**NMR spectra and HPLC chromatograms**

*3,3,3-Trifluoro-2-methylpropyl 4-methylbenzene-1-sulfonate* (***9***)

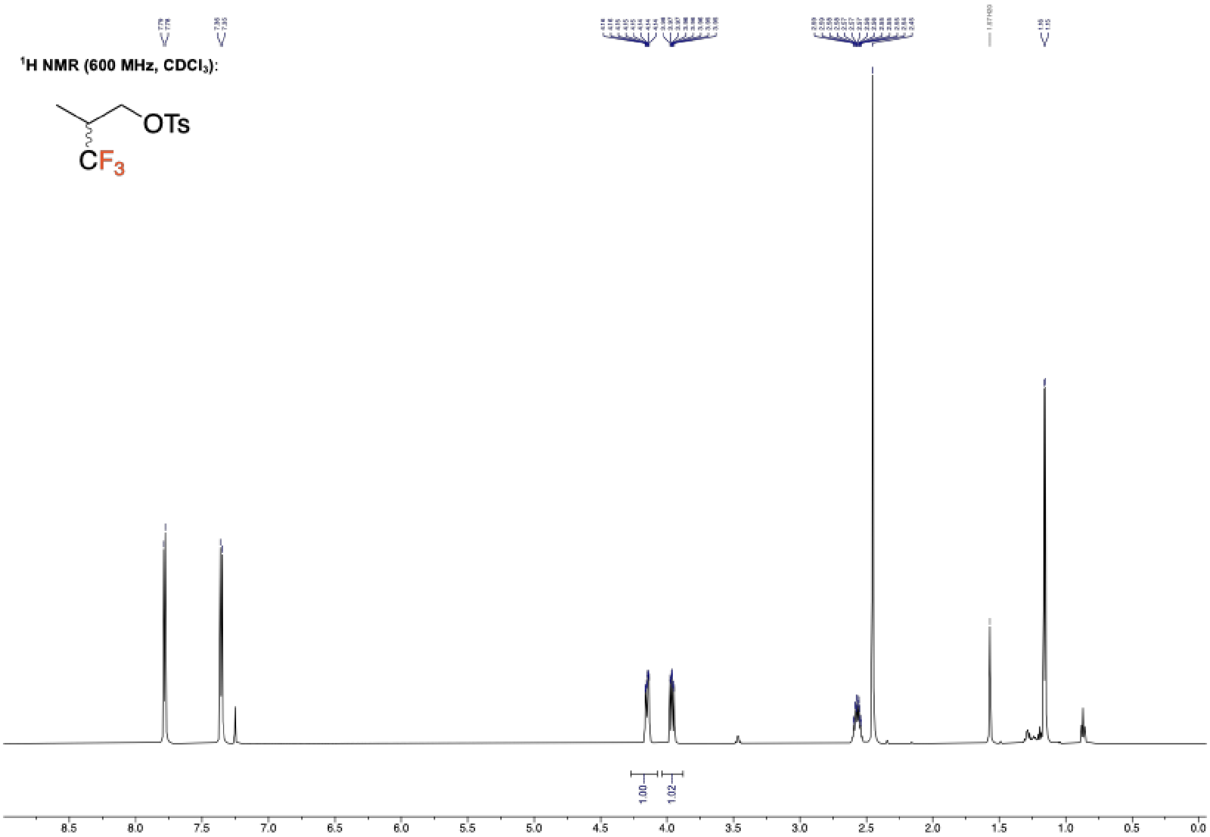


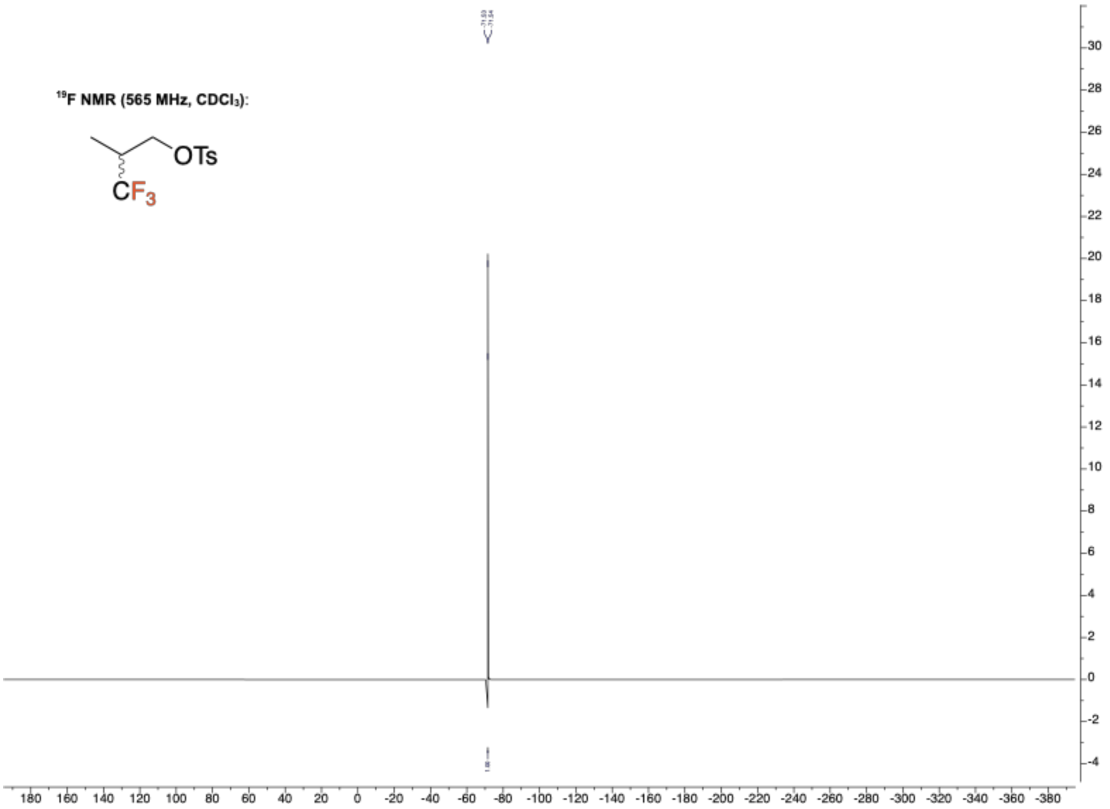

*1,1,1-Trifluoro-3-iodo-2-methylpropane (**10**)*

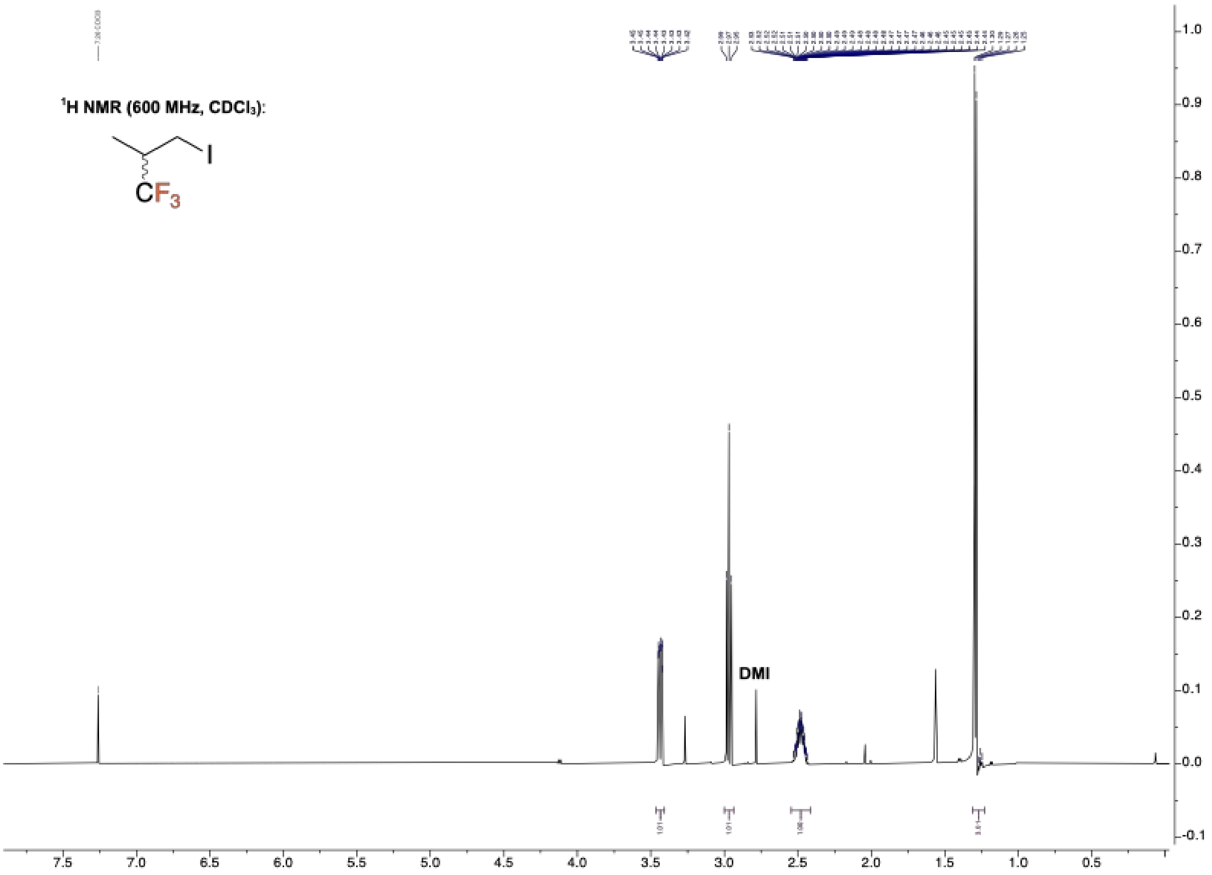


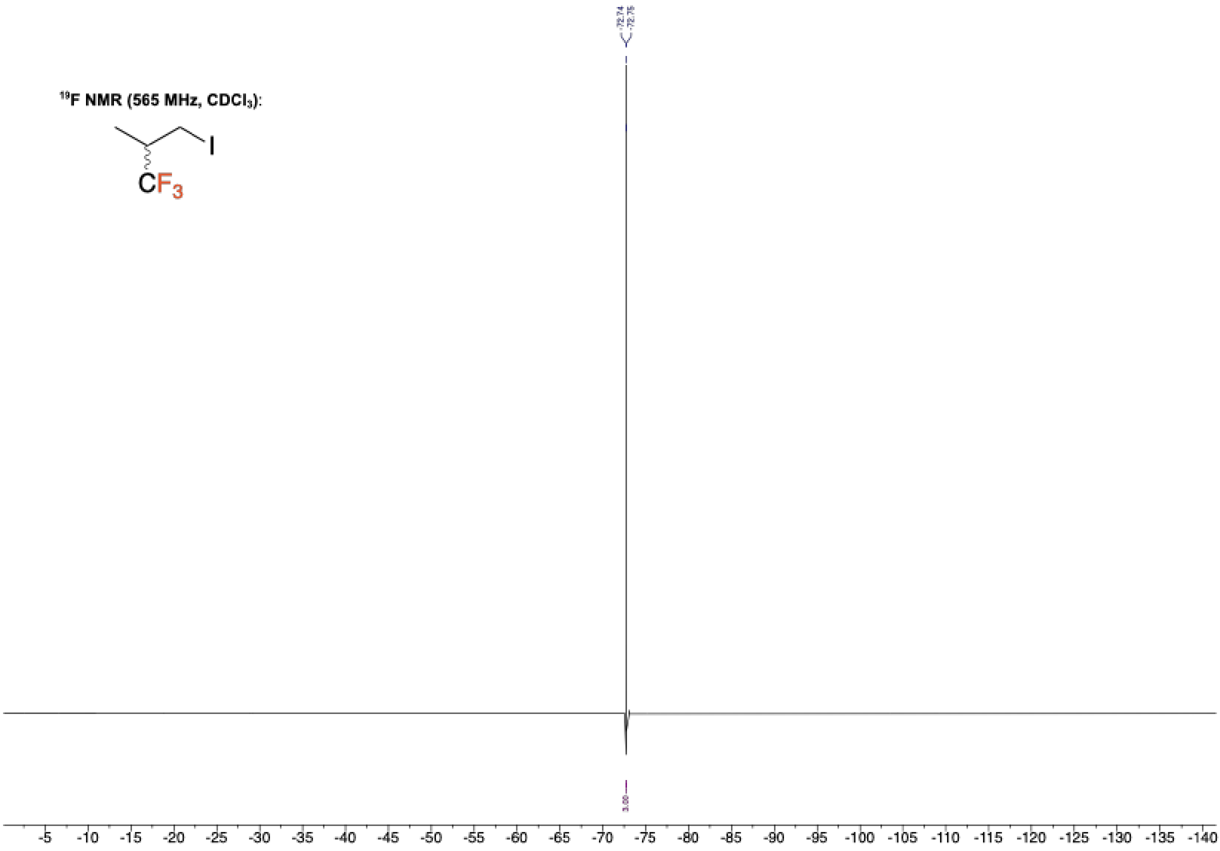

*Ni(II)-Schiff base complex of [2.3.5.6F]TfMePhe* ***6***

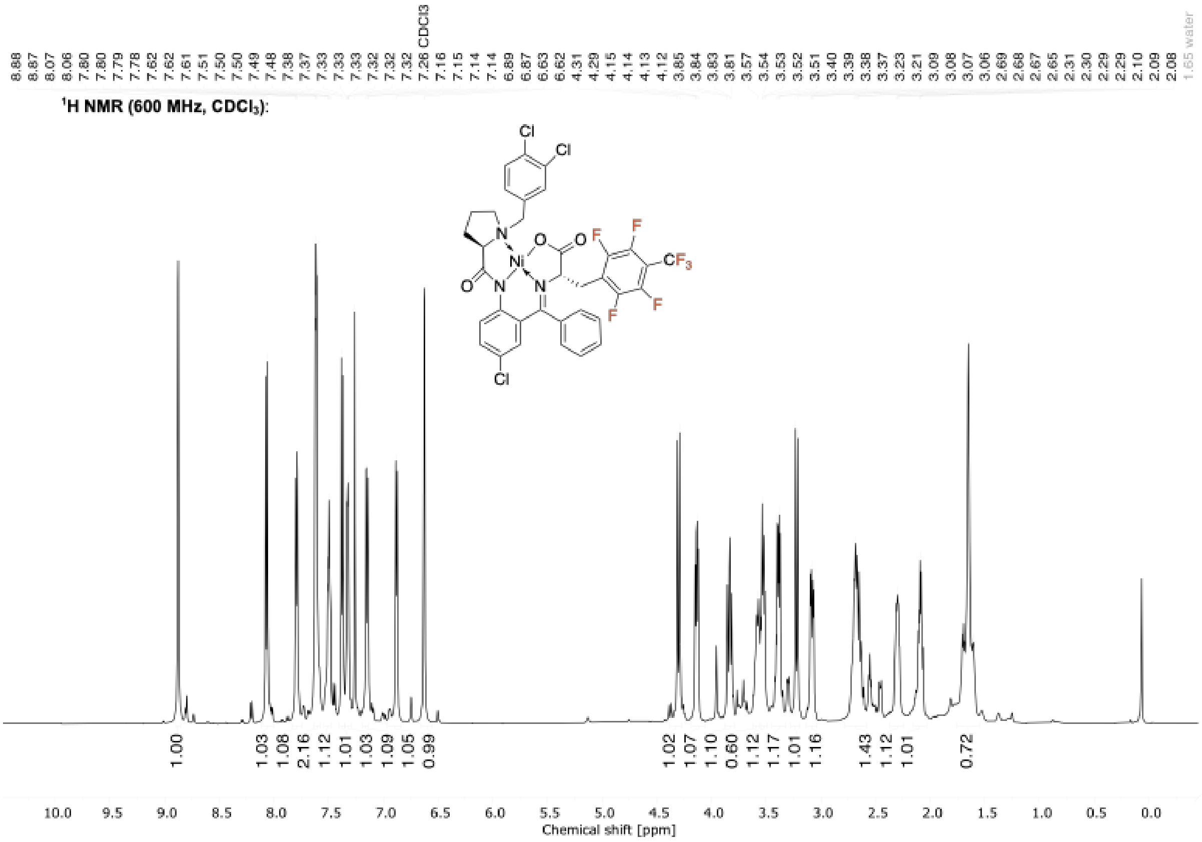


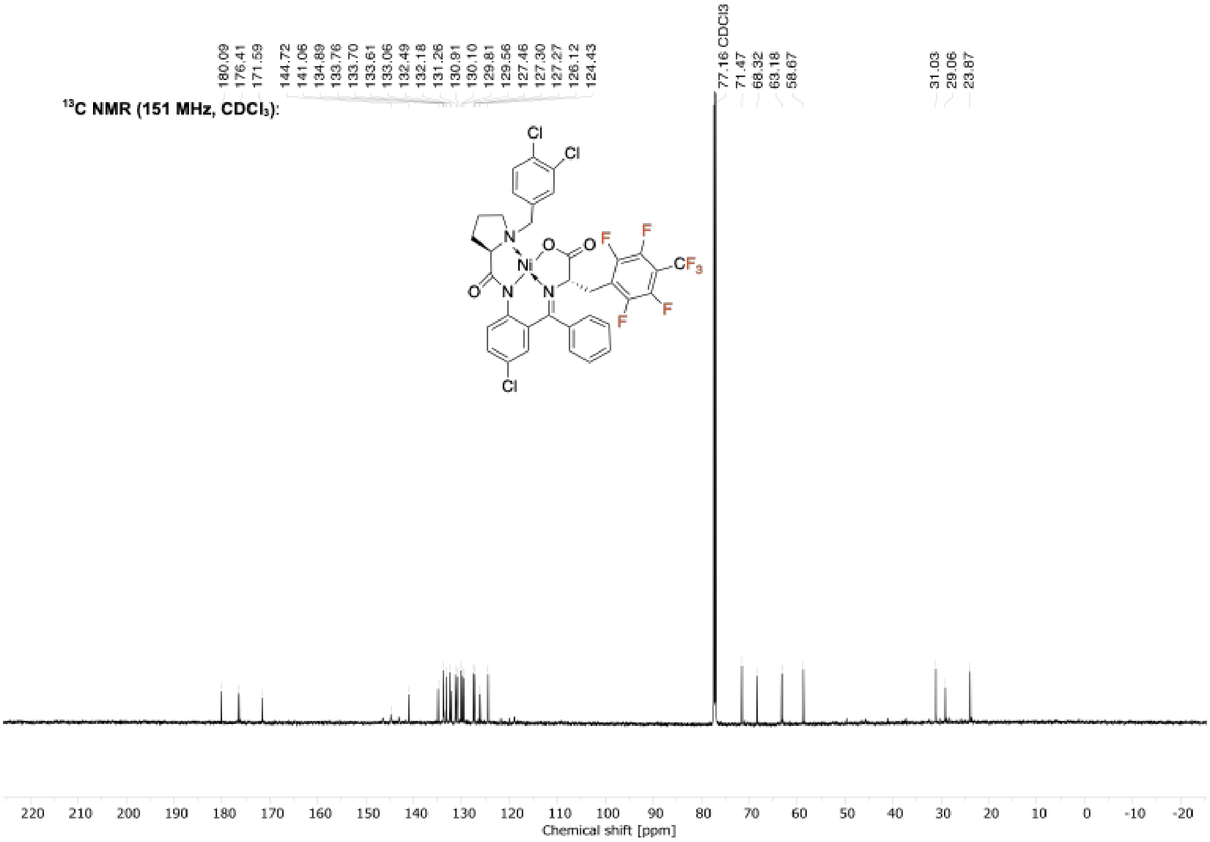

**$^{19}$F NMR (565 MHz, $CDCl_3$):**

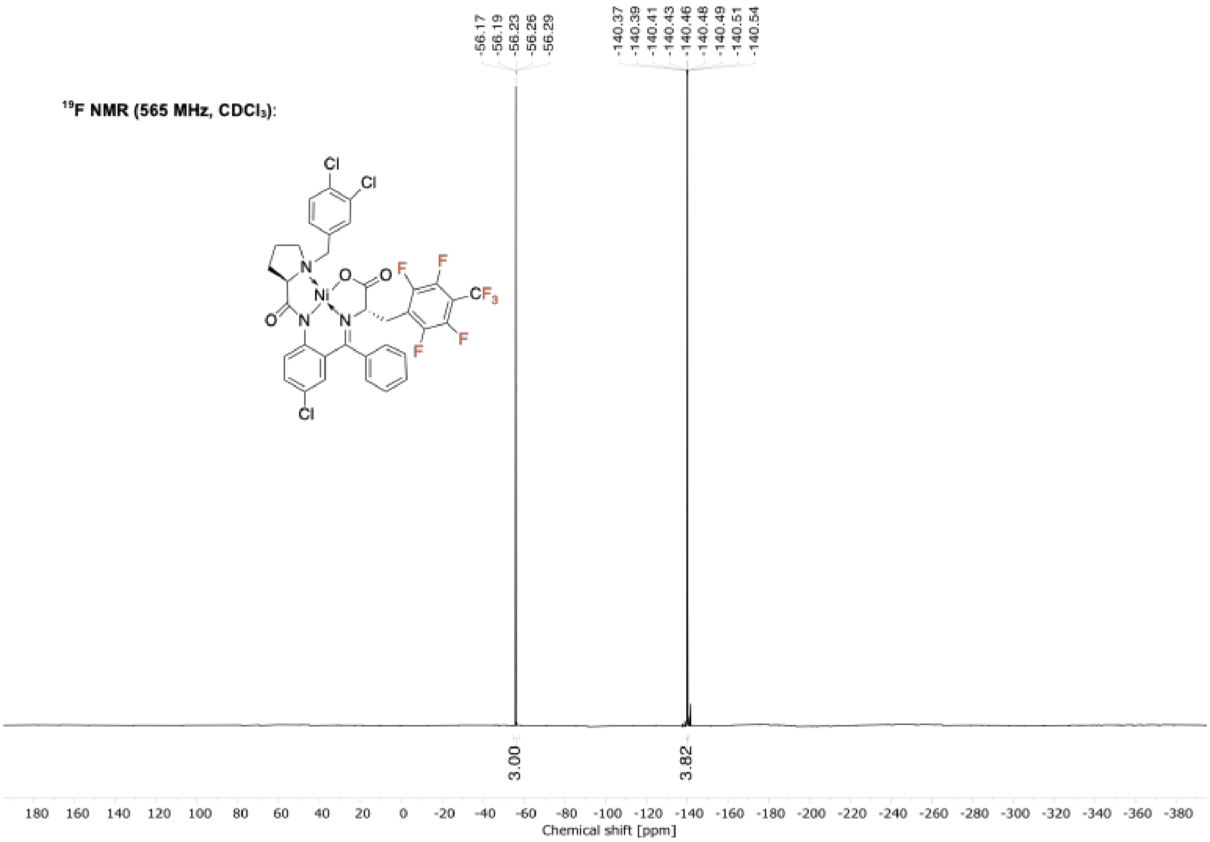

*Ni(II)-Schiff base complex of bisTfMePhe* ***7***

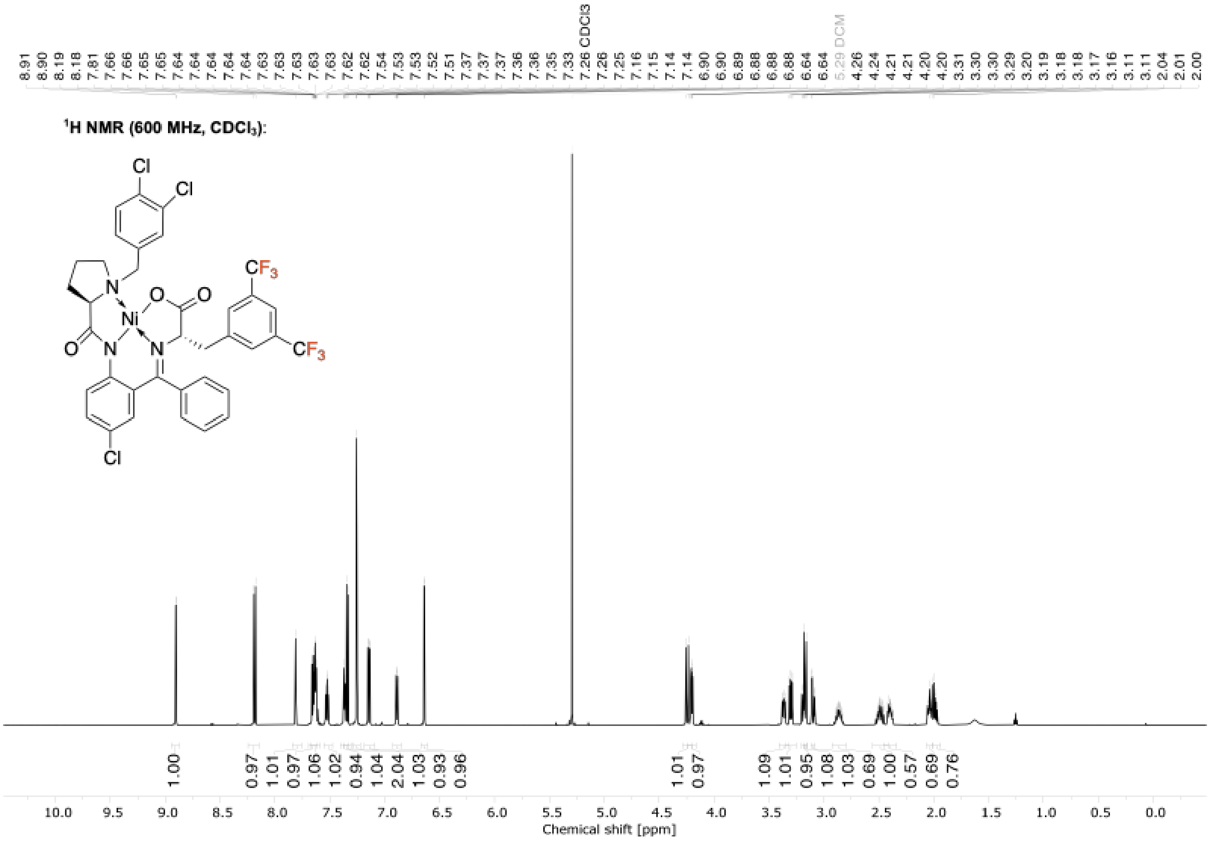


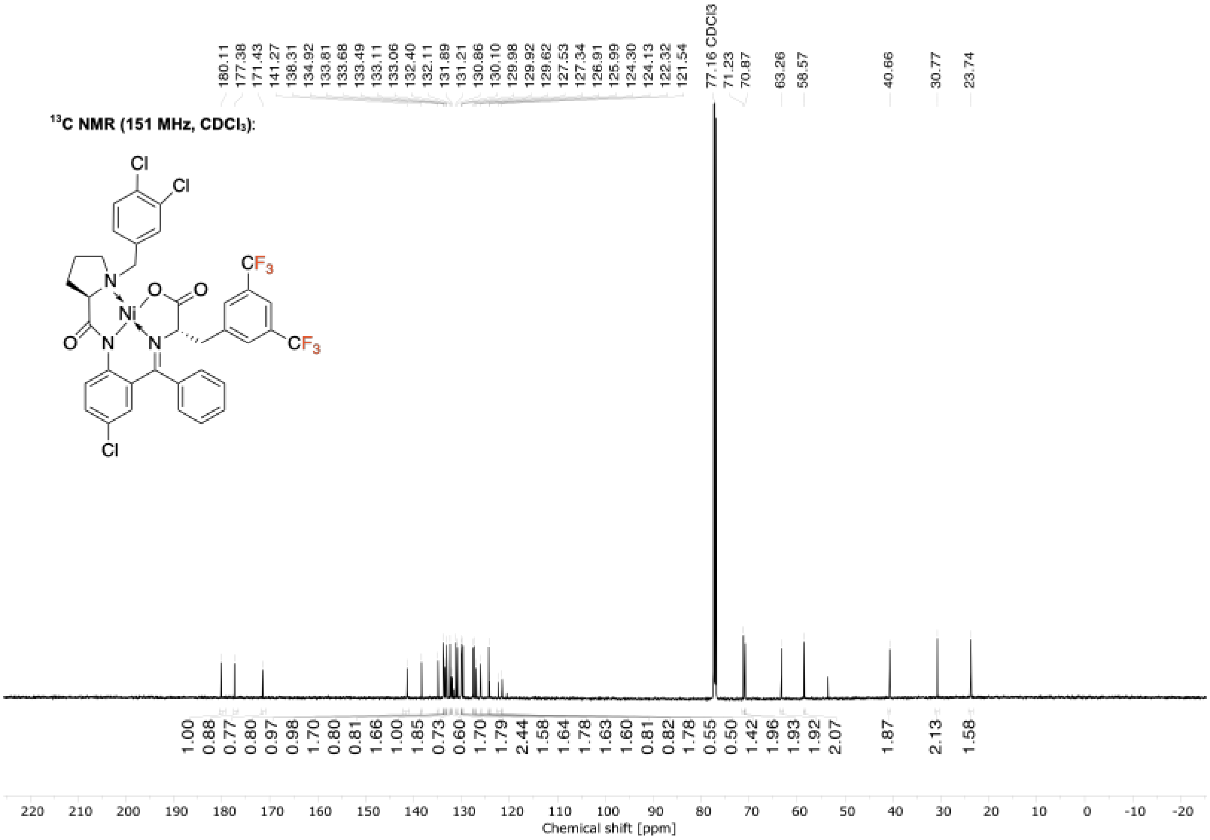

**$^{19}$F NMR (565 MHz, $CDCl_3$):**

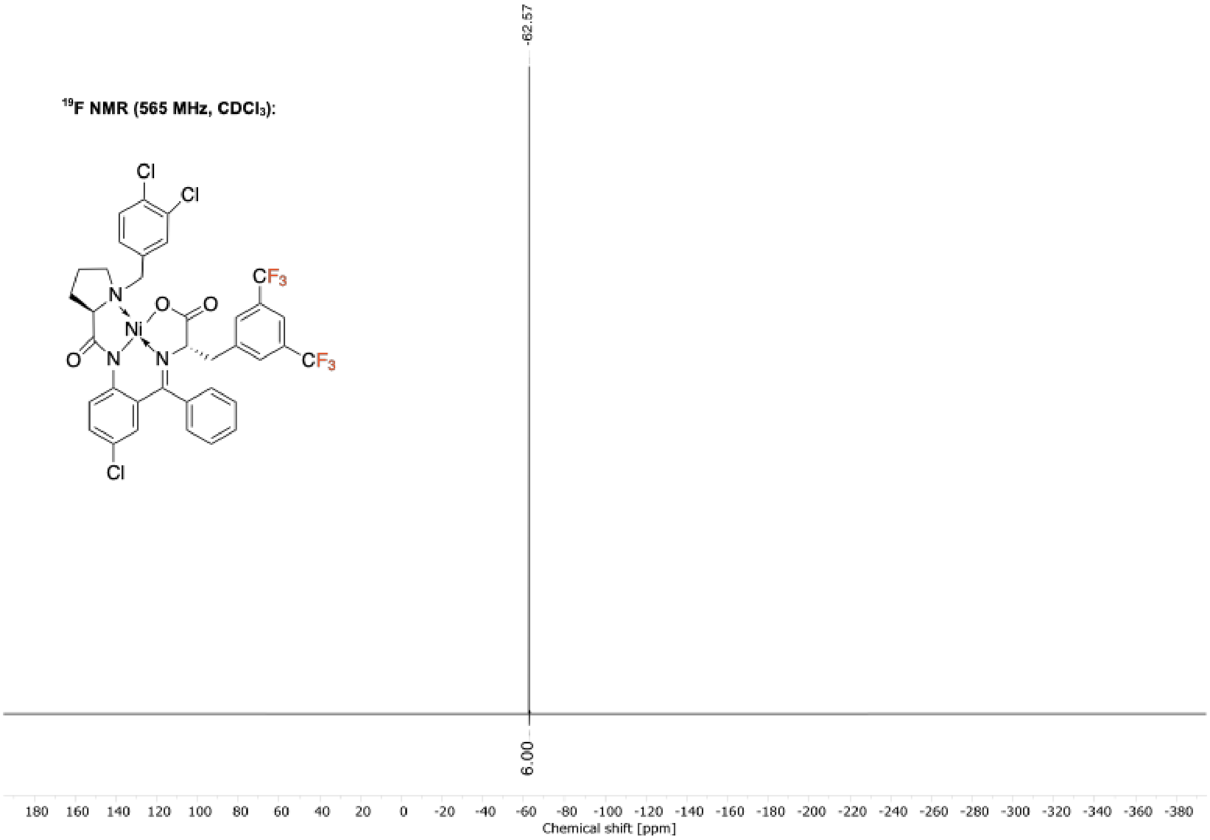

*Ni(II)-Schiff base complexes of (2S,4R)-TfLeu **11a**, (2S,4S)-TfLeu **11b***

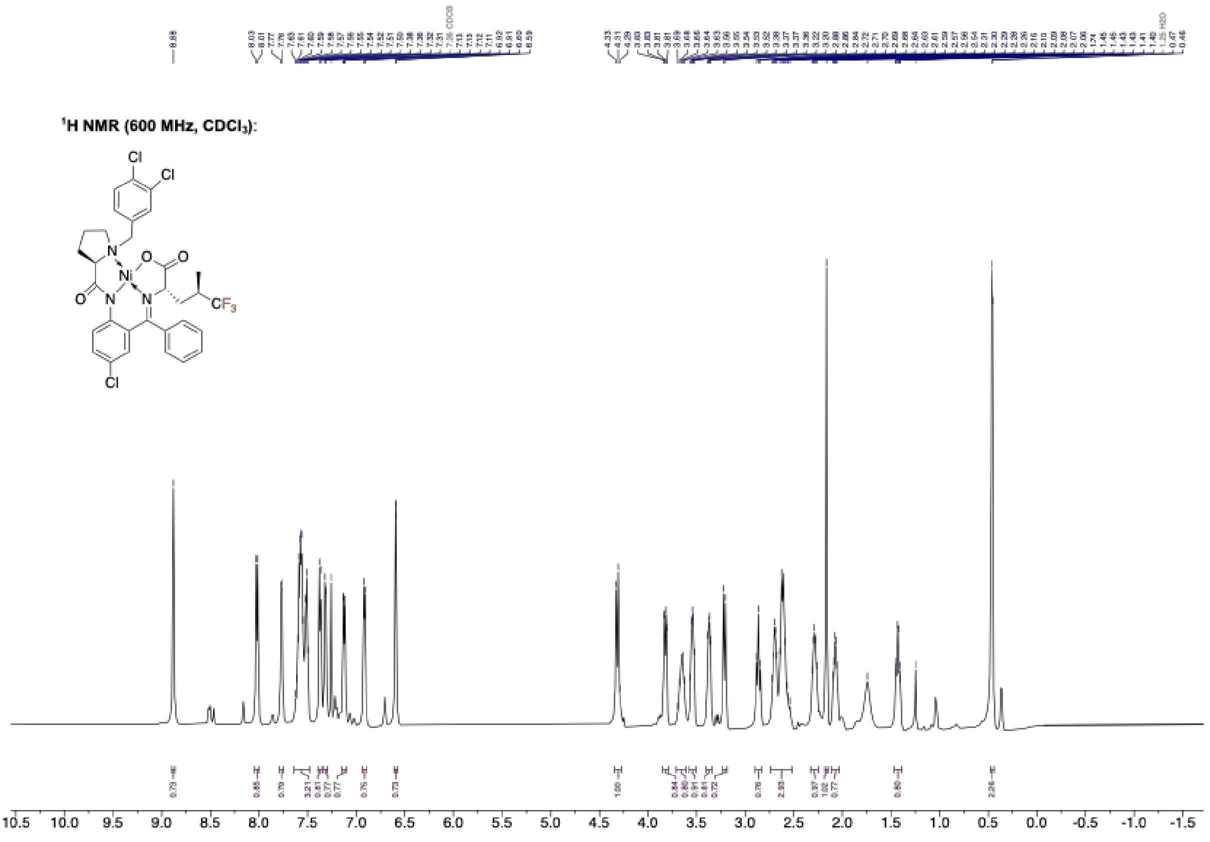


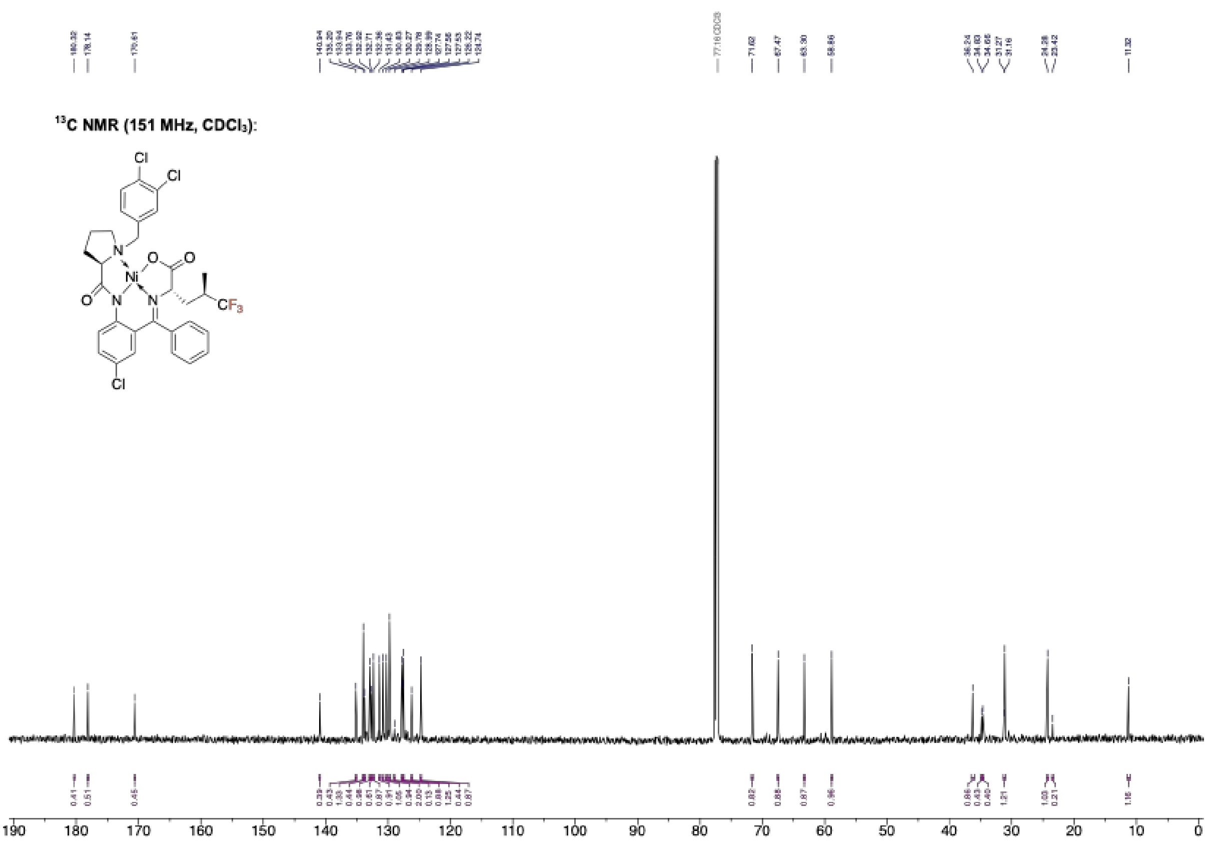

$^{19}$F NMR (565 MHz, $CDCl_3$):

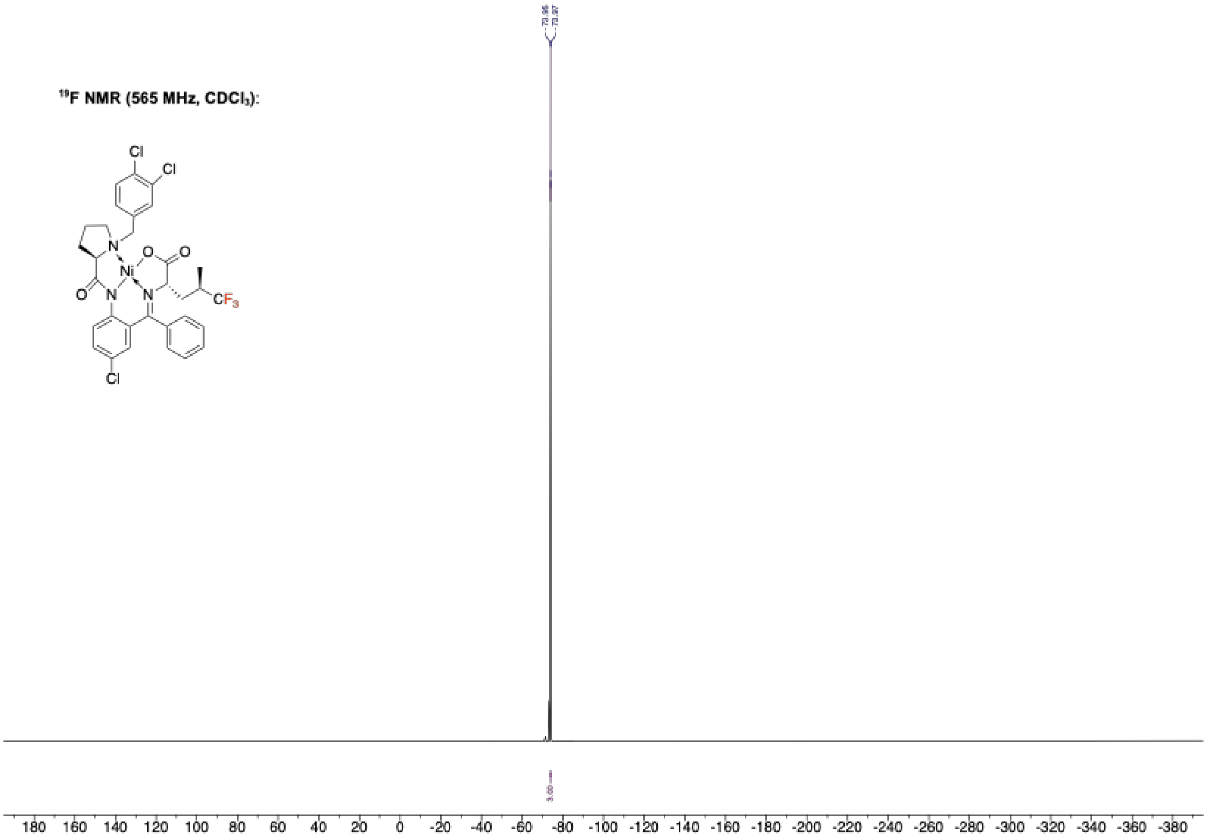

$^{1}$H NMR (600 MHz, $CDCl_3$):

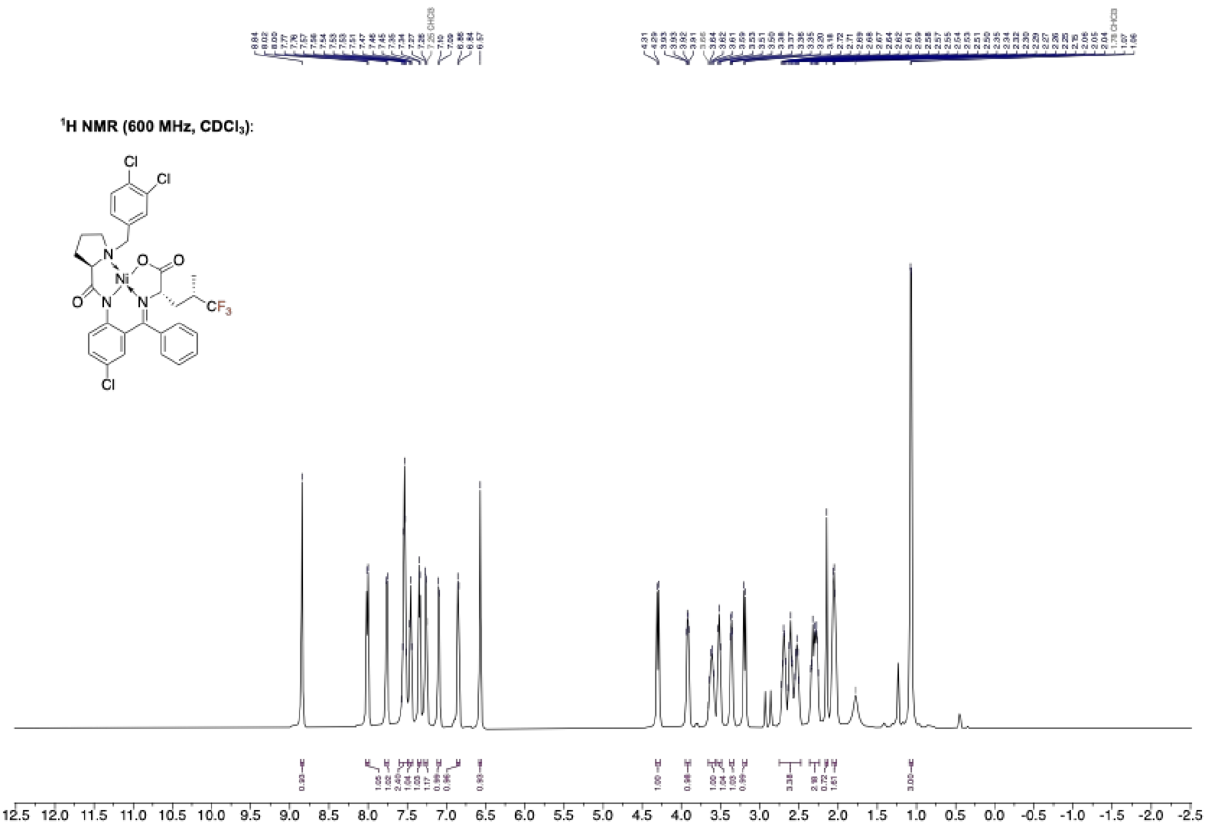

**$^{13}$C NMR (151 MHz, $CDCl_3$):**

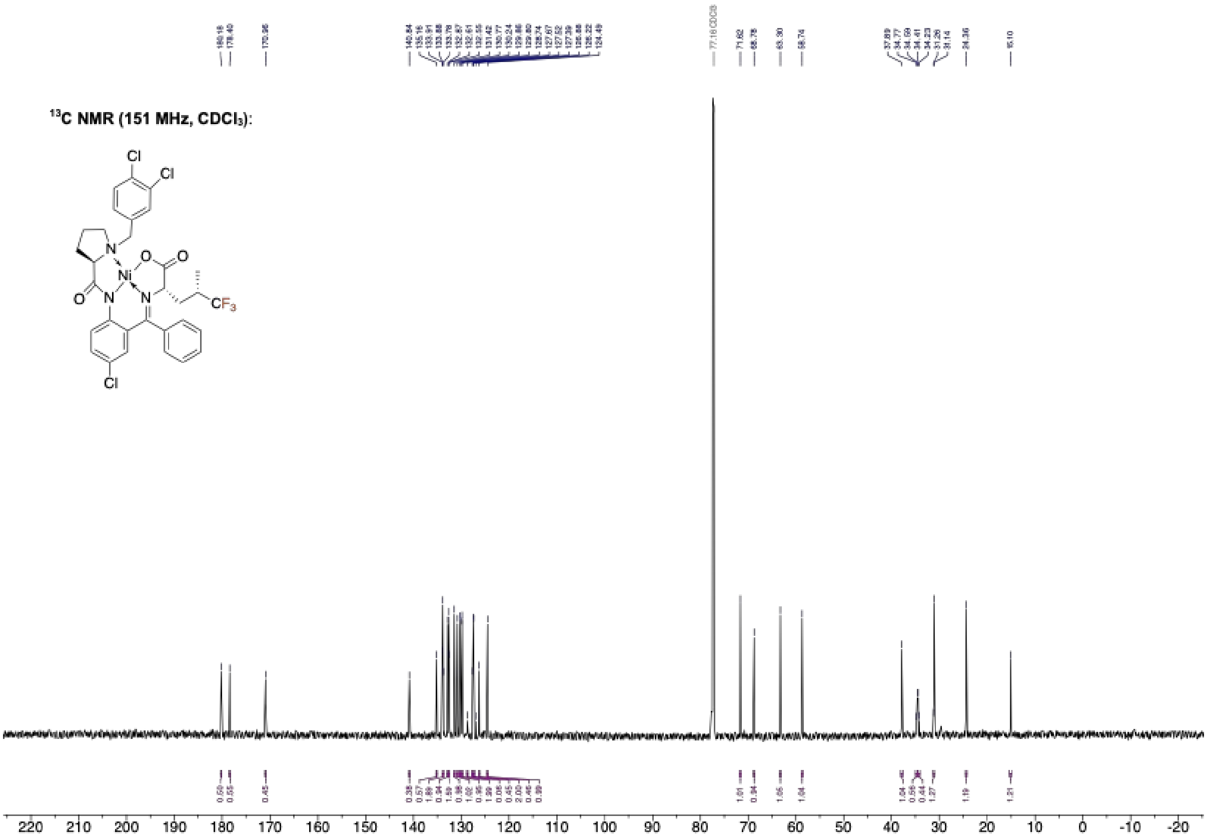


**$^{19}$F NMR (565 MHz, $CDCl_3$):**

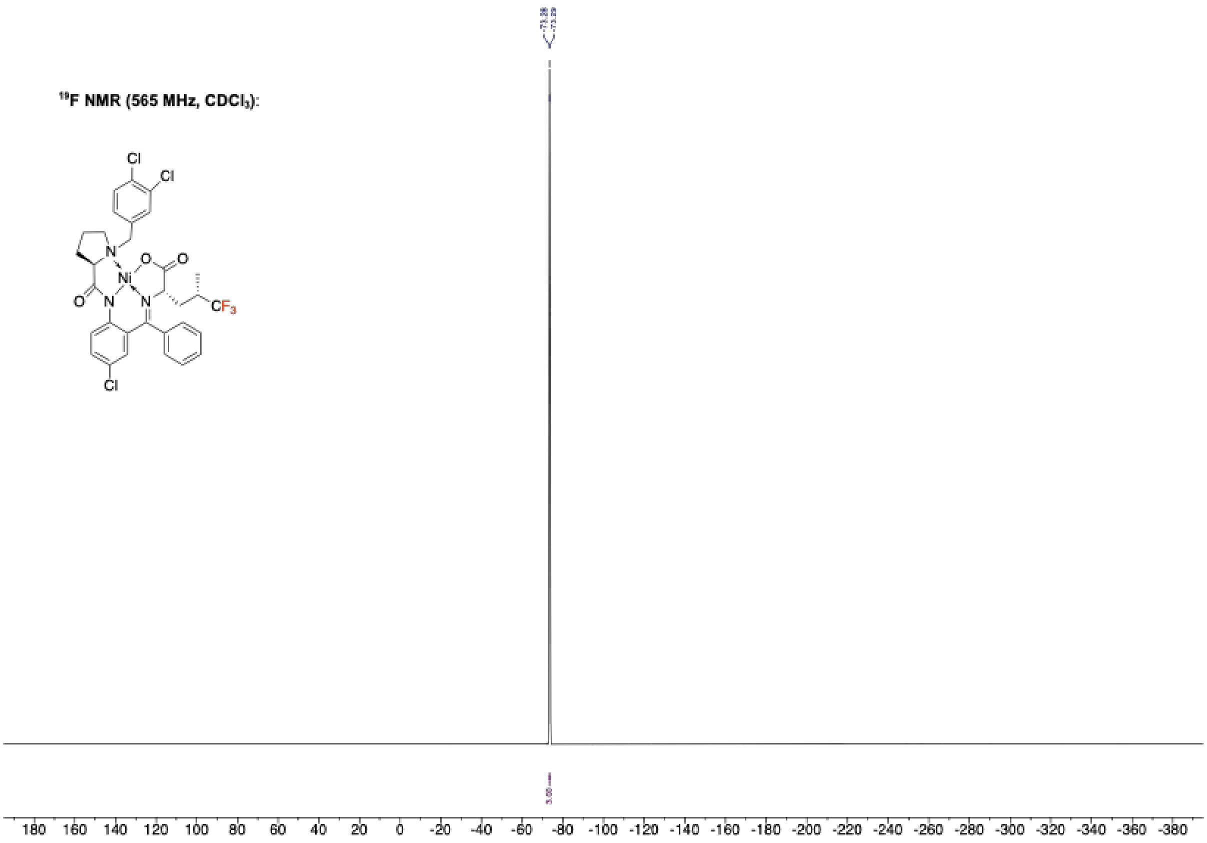

*(S)-2-((((9H-fluoren-9-yl)methoxy)carbonyl)amino)-3-(2,3,5,6-tetrafluoro-4-(tri-fluoromethyl)phenyl)propanoic acid [tetrafluoro(trifluoromethyl)phenylalanine, [2.3.5.6F]TfMePhe]* **2**

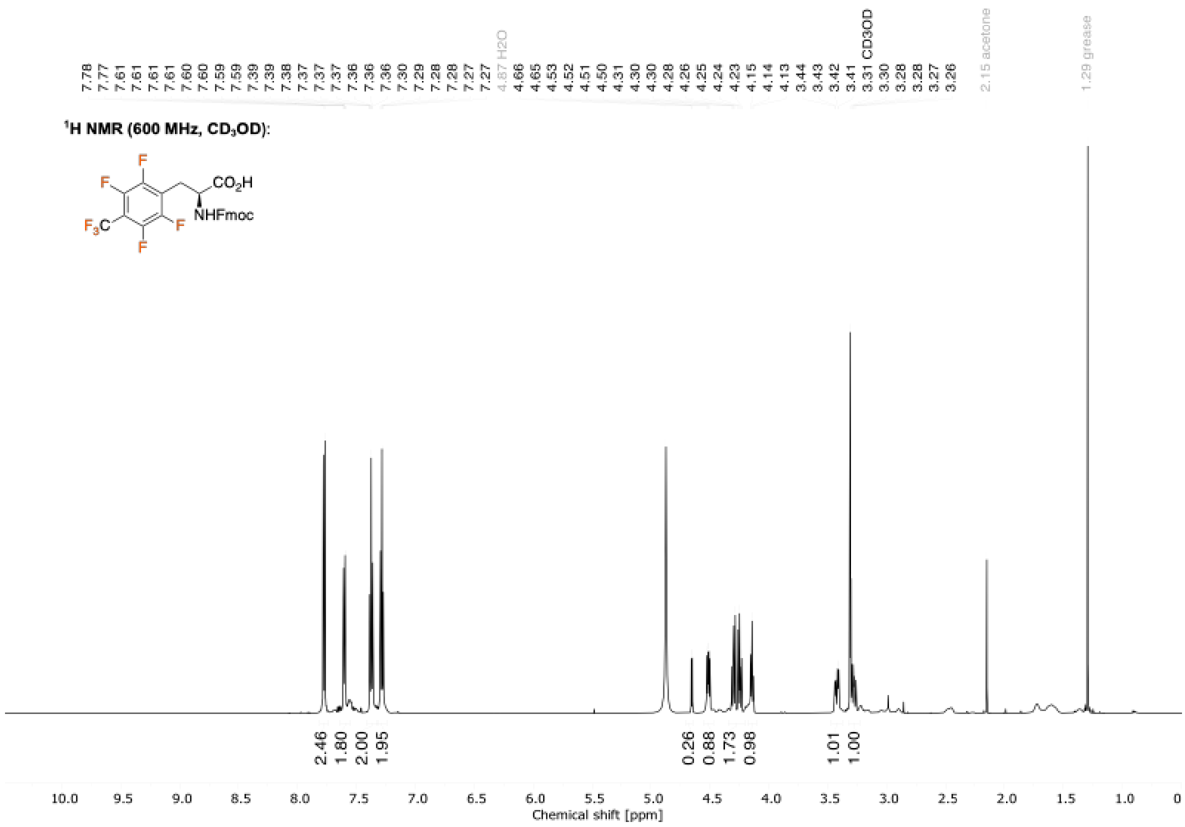


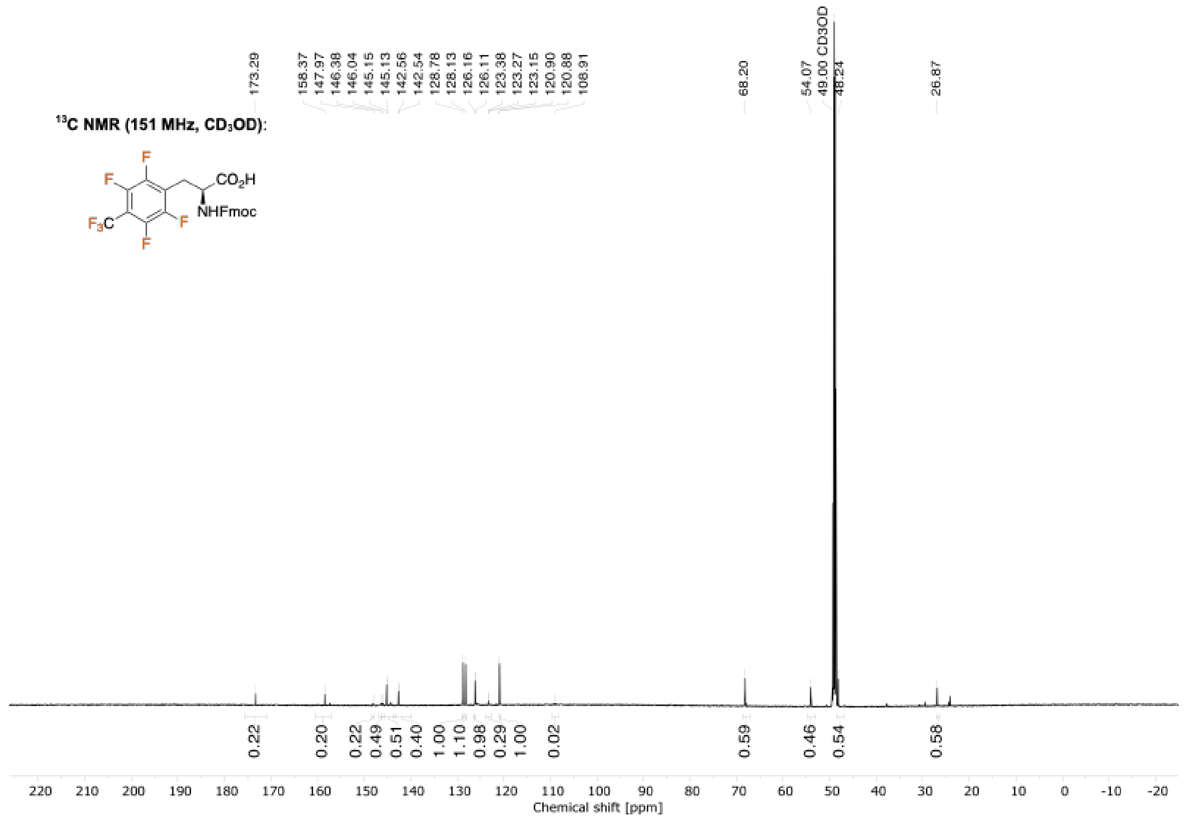

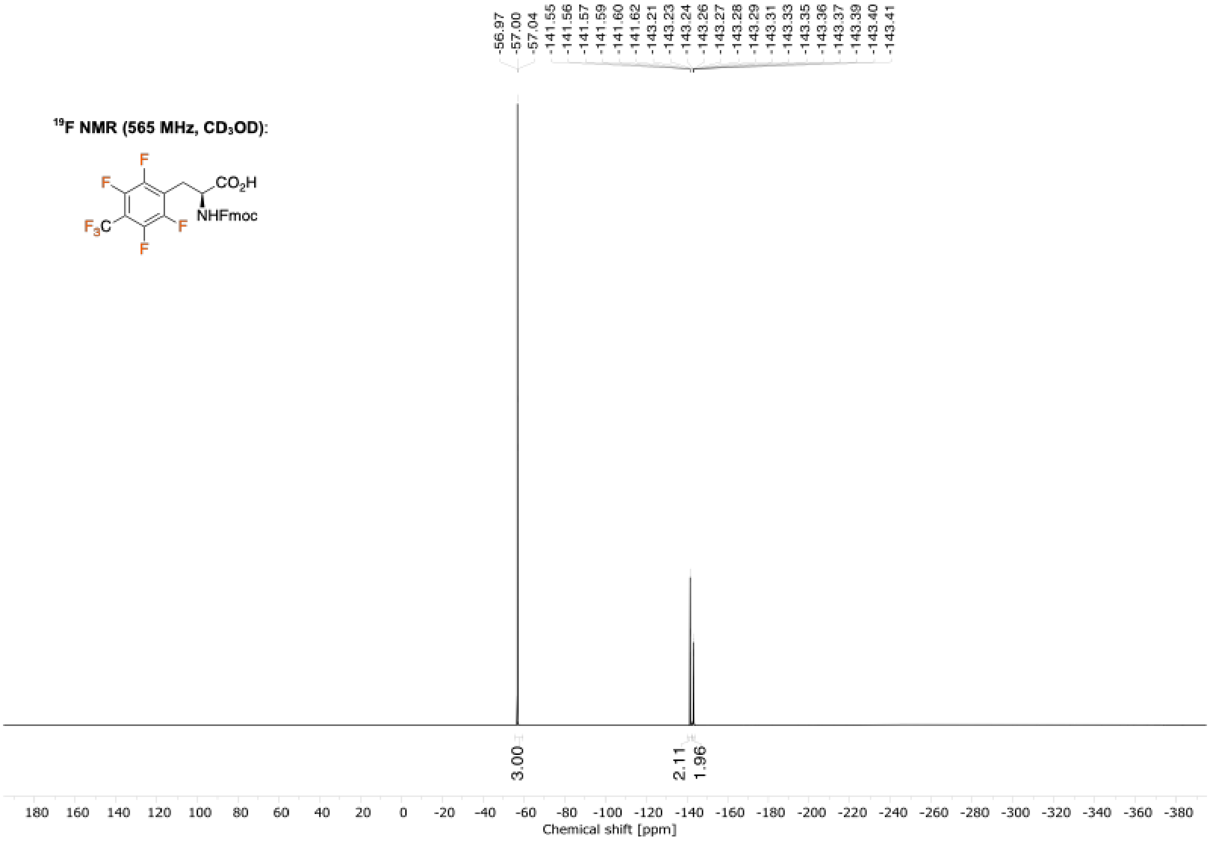

-56.97
-57.00
-57.04
-141.55
-141.56
-141.57
-141.59
-141.60
-141.62
-143.21
-143.23
-143.24
-143.26
-143.27
-143.28
-143.29
-143.31
-143.33
-143.35
-143.36
-143.37
-143.39
-143.40
-143.41
19F NMR (565 MHz, CD3OD):
F
F
CO2H
NHFmoc
F3C
F
F
3.00
2.11
1.96
180 160 140 120 100 80 60 40 20 0 -20 -40 -60 -80 -100 -120 -140 -160 -180 -200 -220 -240 -260 -280 -300 -320 -340 -360 -380
Chemical shift [ppm]

*(S)-2-((((9H-fluoren-9-yl)methoxy)carbonyl)amino)-3-(3,5-bis(trifluoromethyl) phenyl)propanoic acid [bis(trifluoromethyl)phenylalanine, bisTfMePhe]* ***3***

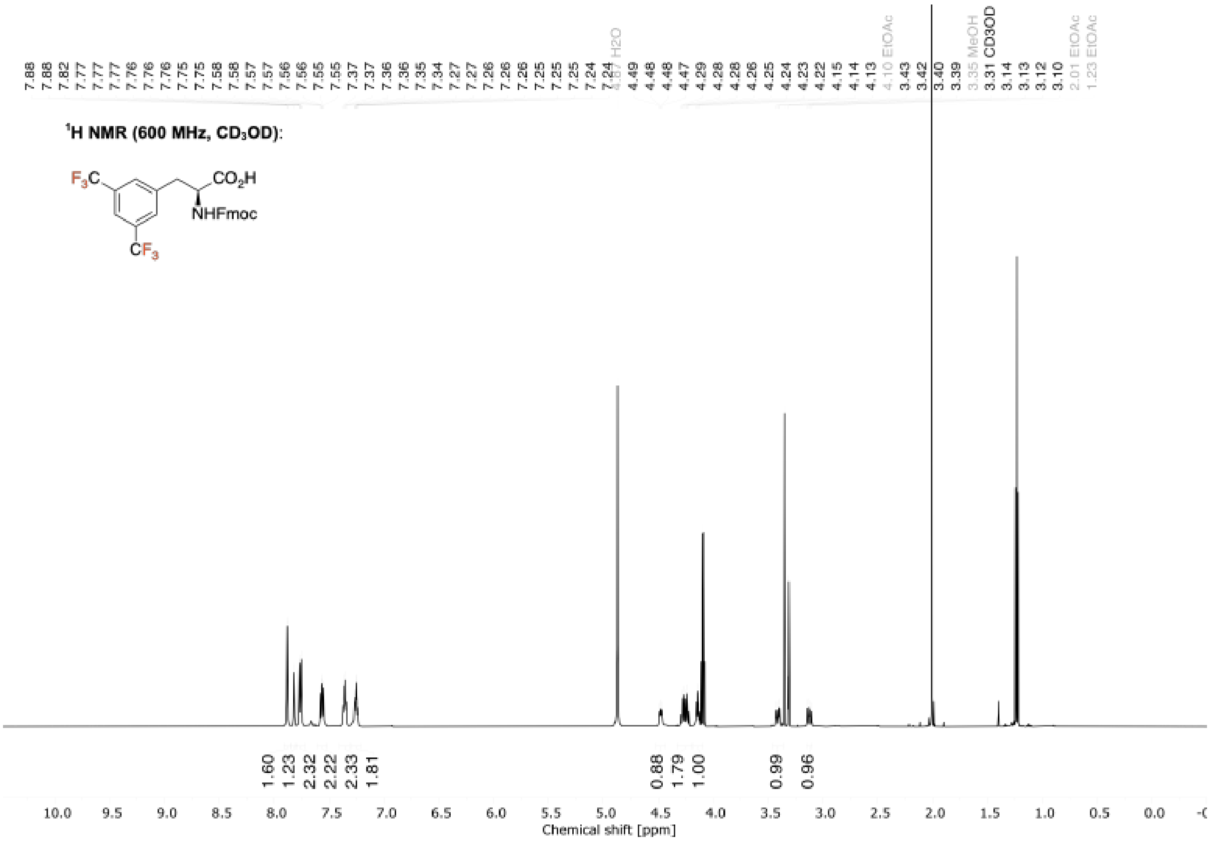


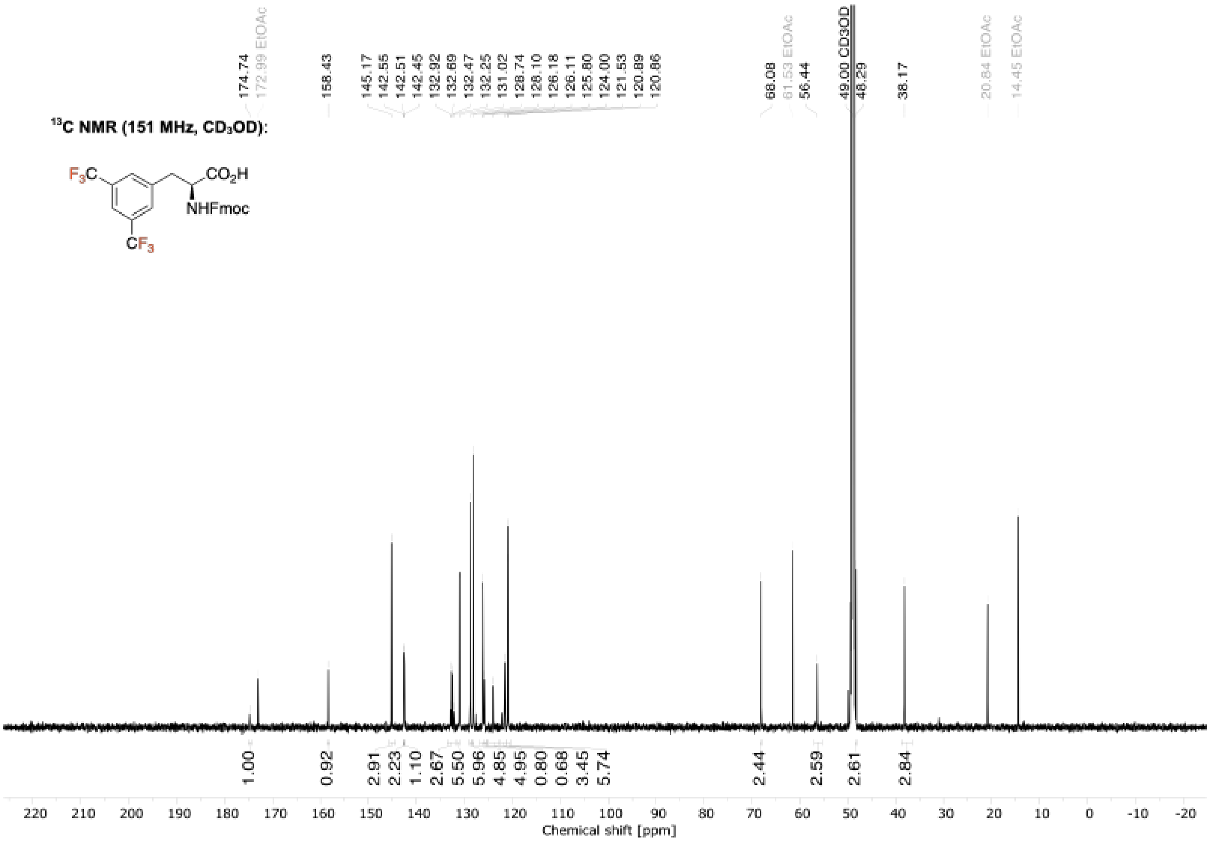

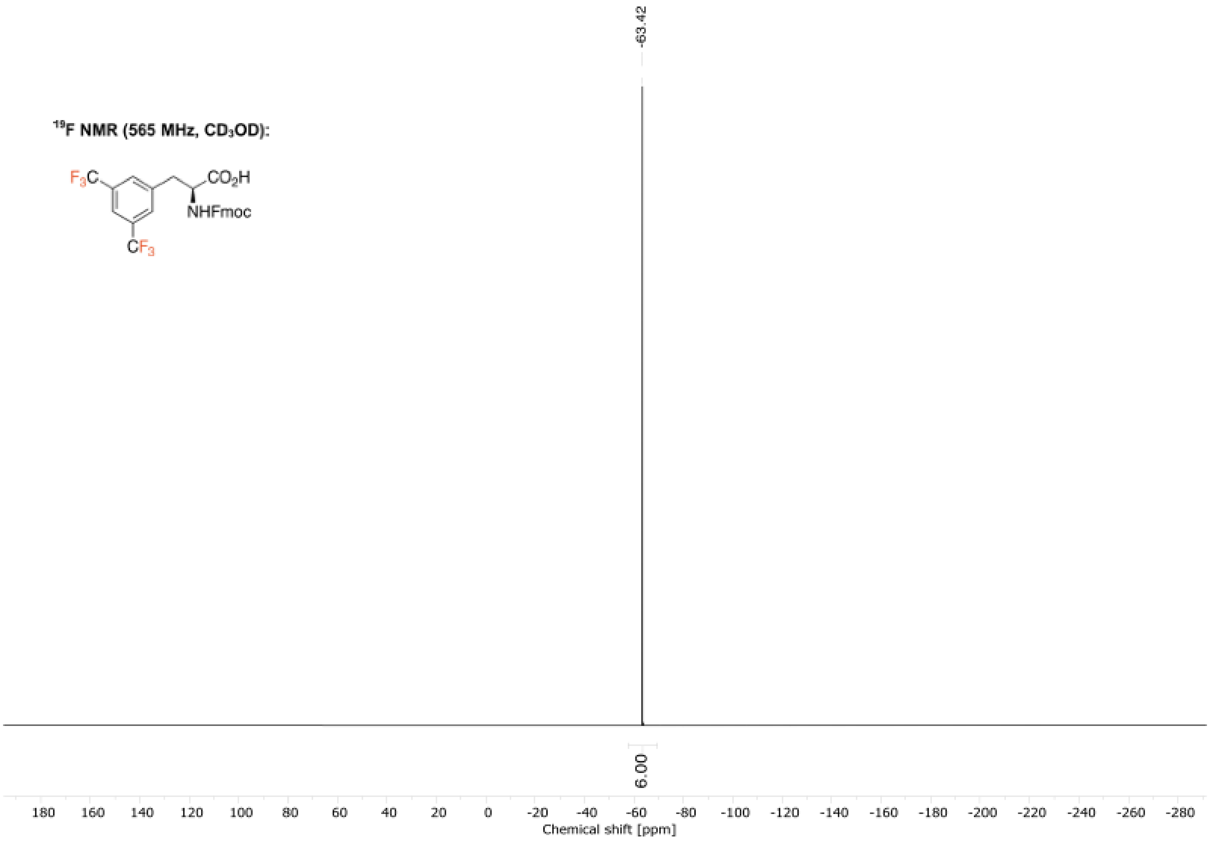
-63.42
19F NMR (565 MHz, CD3OD):
F3C
CO2H
NHFmoc
CF3
6.00
180 160 140 120 100 80 60 40 20 0 -20 -40 -60 -80 -100 -120 -140 -160 -180 -200 -220 -240 -260 -280
Chemical shift [ppm]

*(2S,4R)-2-((((9H-fluoren-9-yl)methoxy)carbonyl)amino)-5,5,5-trifluoro-4-methylpentanoic acid [Trifluoroleucine, TfLeu]* ***12a***

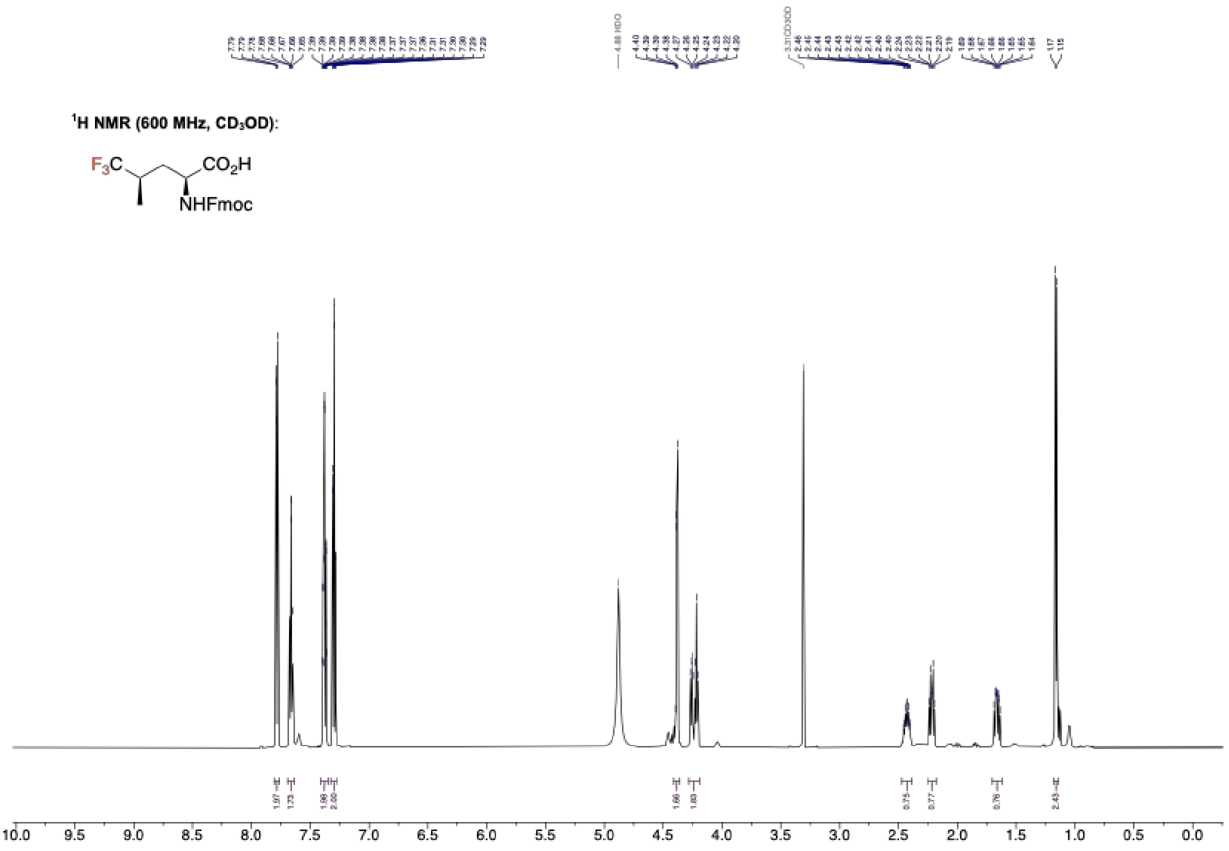


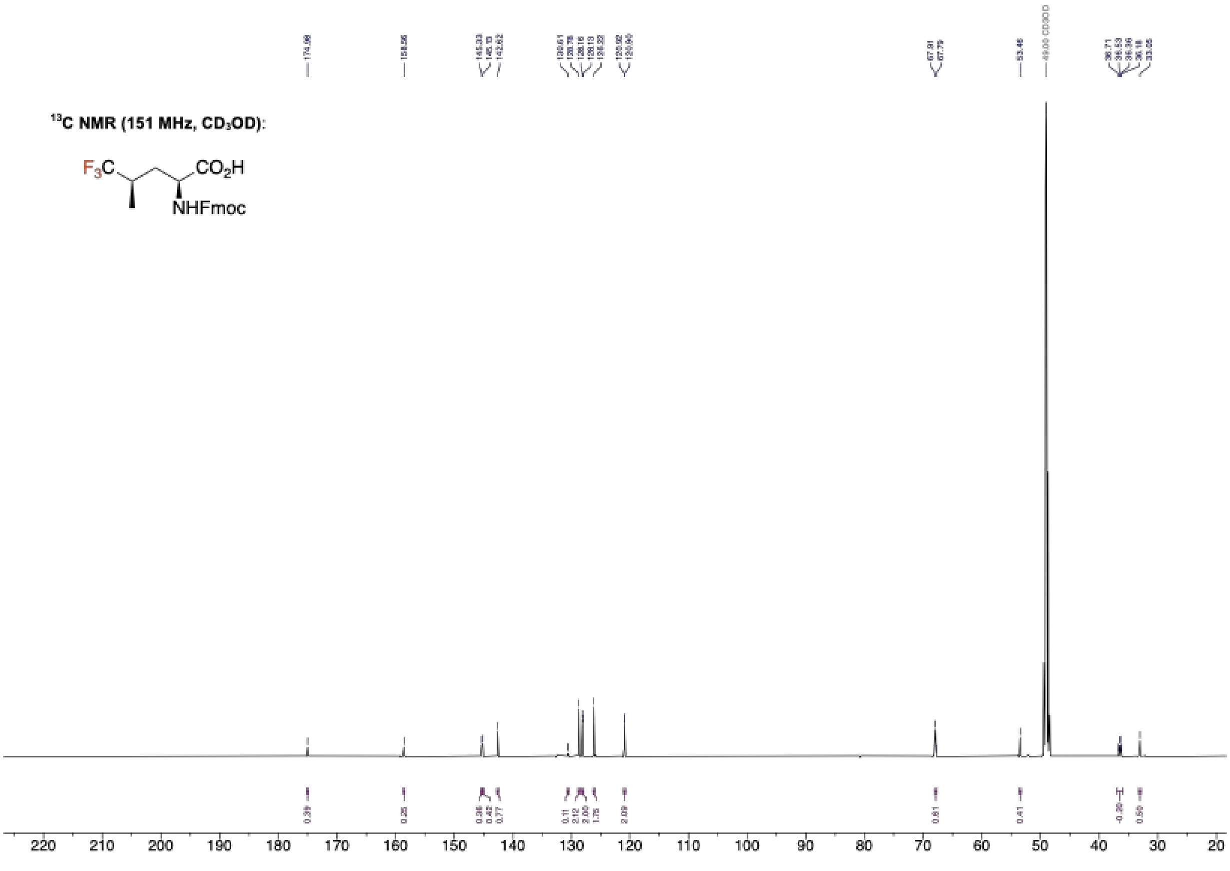

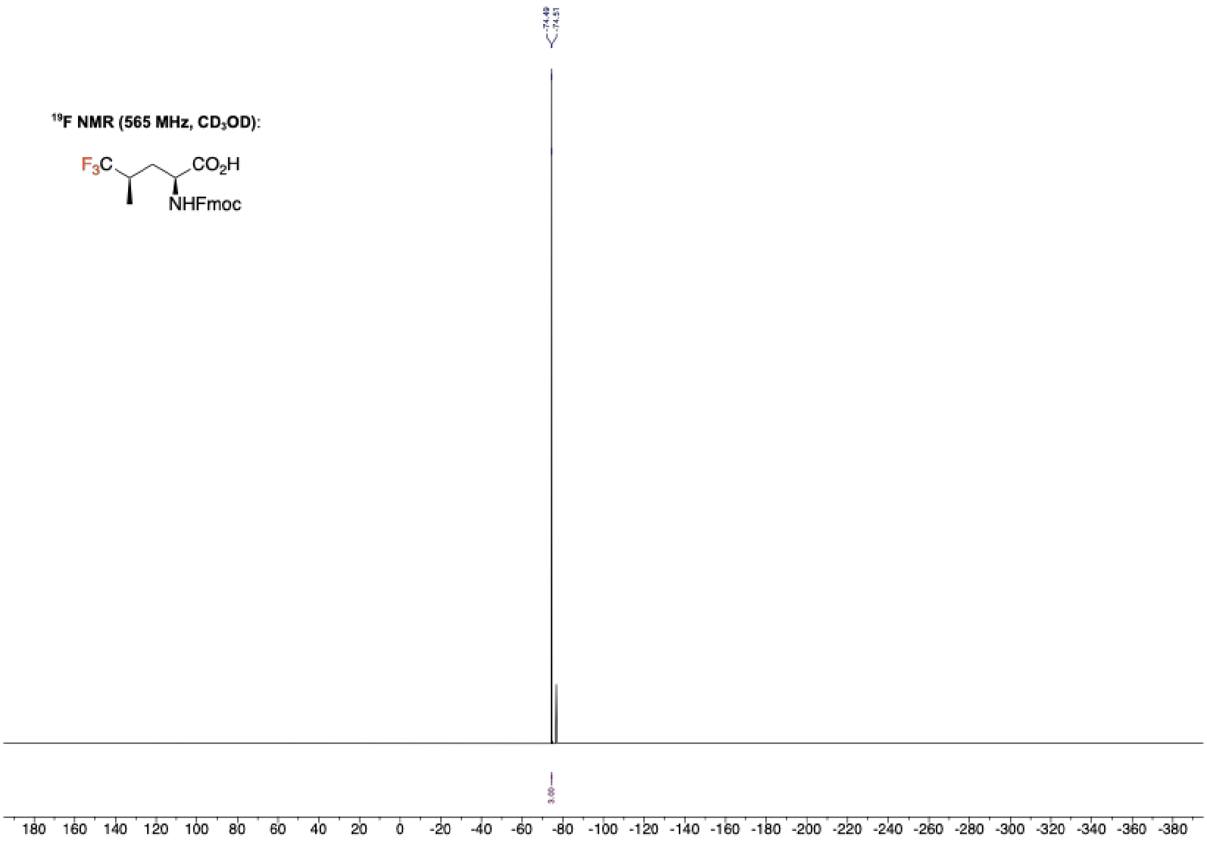

-74.49
-74.51
19F NMR (565 MHz, CD3OD):
F3C
CO2H
NHFmoc
3.00
180 160 140 120 100 80 60 40 20 0 -20 -40 -60 -80 -100 -120 -140 -160 -180 -200 -220 -240 -260 -280 -300 -320 -340 -360 -380

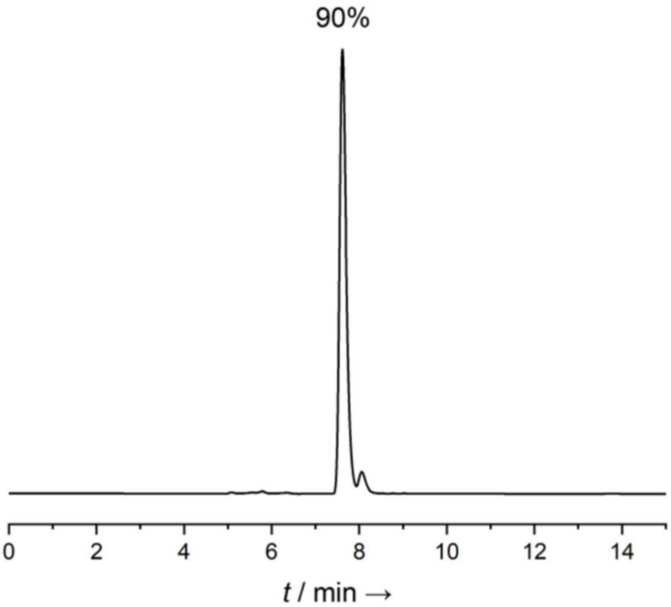


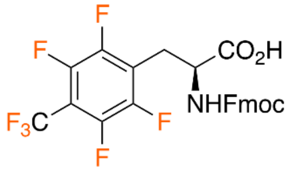


Chiral HPLC, Method:

MeCN:MeOH:$H_2O$ = 148:850:2, 15 min

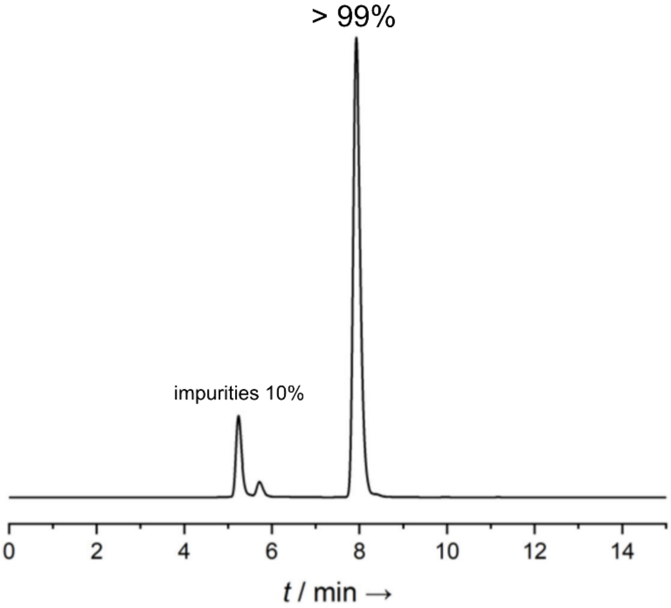


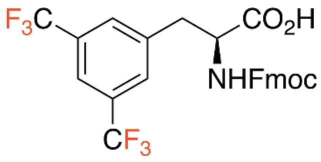


Chiral HPLC, Method:

MeCN:MeOH:$H_2O$ = 148:850:2, 15 min

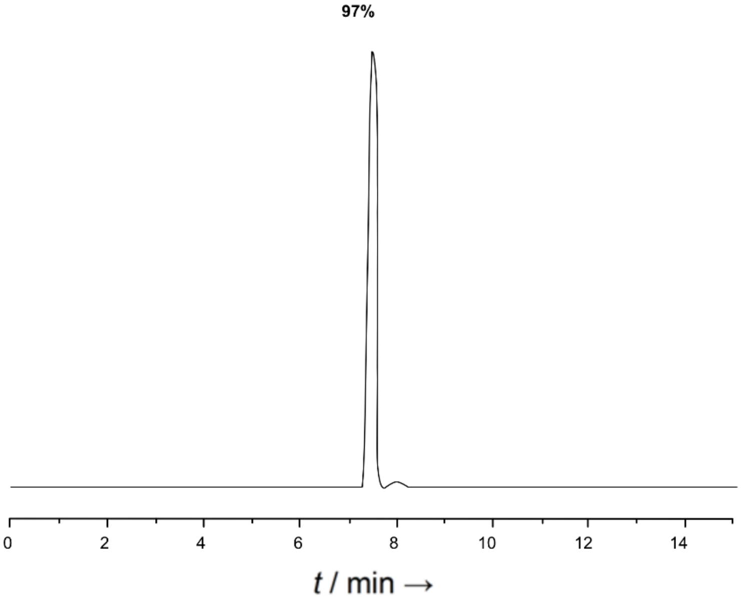


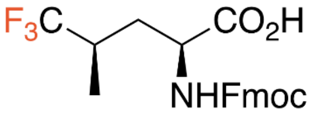


Chiral HPLC, Method:

MeCN:MeOH:$H_2O$ = 148:850:2, 15 min

In order to validate the separation efficiency of the HPLC method to determine the enantiomeric purity of the fluorinated amino acids in our previous publication racemic mixtures of the corresponding fluorinated amino acids were prepared by a procedure described by Yamada et al. and subsequently analyzed [1].

## References

1. Yamada, S.H., C.; Yoshioka, R.; Chibata, I. , *Method for the racemization of optically active amino acids.* Journal of Organic Chemistry, 1983. **48**(6): p. 843-846.
